# Metabolic Sensing of Extracytoplasmic Copper Availability via Translational Control by a Nascent Exported Protein

**DOI:** 10.1128/mbio.03040-22

**Published:** 2023-01-04

**Authors:** Yavuz Öztürk, Andreea Andrei, Crysten E. Blaby-Haas, Noel Daum, Fevzi Daldal, Hans-Georg Koch

**Affiliations:** a Institut für Biochemie und Molekularbiologie, ZBMZ, Faculty of Medicine, Albert-Ludwigs-Universität Freiburg, Freiburg, Germany; b Fakultät für Biologie, Albert-Ludwigs-Universität Freiburg, Freiburg, Germany; c Department of Energy Joint Genome Institute, Lawrence Berkeley National Laboratory, Berkeley, California, USA; d Molecular Foundry, Lawrence Berkeley National Laboratory, Berkeley, California, USA; e Department of Biology, University of Pennsylvania, Philadelphia, Pennsylvania, USA; University of Arizona

**Keywords:** *Rhodobacter capsulatus*, metabolite sensing, copper homeostasis, translational control, multicopper oxidase

## Abstract

Metabolic sensing is a crucial prerequisite for cells to adjust their physiology to rapidly changing environments. In bacteria, the response to intra- and extracellular ligands is primarily controlled by transcriptional regulators, which activate or repress gene expression to ensure metabolic acclimation. Translational control, such as ribosomal stalling, can also contribute to cellular acclimation and has been shown to mediate responses to changing intracellular molecules. In the current study, we demonstrate that the cotranslational export of the Rhodobacter capsulatus protein CutF regulates the translation of the downstream *cutO*-encoded multicopper oxidase CutO in response to extracellular copper (Cu). Our data show that CutF, acting as a Cu sensor, is cotranslationally exported by the signal recognition particle pathway. The binding of Cu to the periplasmically exposed Cu-binding motif of CutF delays its cotranslational export via its C-terminal ribosome stalling-like motif. This allows for the unfolding of an mRNA stem-loop sequence that shields the ribosome-binding site of *cutO*, which favors its subsequent translation. Bioinformatic analyses reveal that CutF-like proteins are widely distributed in bacteria and are often located upstream of genes involved in transition metal homeostasis. Our overall findings illustrate a highly conserved control mechanism using the cotranslational export of a protein acting as a sensor to integrate the changing availability of extracellular nutrients into metabolic acclimation.

## INTRODUCTION

Living cells have evolved complex sensing and signaling systems for ensuring survival in rapidly changing environments. This is particularly important for single-cell organisms, such as bacteria. They engage multiple sensing systems in parallel, including transcriptional regulators (1), two-component systems (2), and chemotactic responses (3). Metabolite sensing by these systems guarantees a sufficient nutrient supply while simultaneously helping to evade potentially toxic compounds. However, the situation is more complex for micronutrients, which are essential for cell metabolism but could be toxic, even at low concentrations. This is exemplified by the multifaceted bacterial response to variations in the concentration of copper (Cu) (4).

Cu is an essential micronutrient that is used as a cofactor by cuproenzymes, such as cytochrome oxidases (Cox) or nitrous oxide reductases (4–6). However, free Cu is also toxic due to its redox properties, as it facilitates the formation of reactive oxygen species, interferes with thiol groups in proteins, and damages various metalloproteins (6, 7). Consequently, no free Cu is detectable in the bacterial cytoplasm (8), with Cu homeostasis being achieved by an intricate network of Cu transporters and Cu chaperones. This ensures a sufficient Cu supply for cuproprotein biogenesis while preventing the accumulation of free Cu (4, 9–12). Periplasmic multicopper oxidases provide a further line of defense against Cu toxicity by converting Cu(I) to the less toxic Cu(II) in the Gram-negative, purple nonsulfur, facultative photosynthetic bacterium Rhodobacter capsulatus (13–15).

The transcriptional activation of genes encoding Cu-exporting proteins and Cu-chaperones represent the main bacterial response to increased Cu concentrations (16–18). MerR-like transcriptional activators, such as CueR, sense Cu in the cytoplasm and regulate the production of the cytosolic Cu chaperone CopZ, the Cu-exporting P_1B_-type ATPase CopA, and the multicopper oxidase CutO (19–21). Transcriptional repressors also regulate Cu-dependent gene expression. At low cytoplasmic Cu concentrations, repressors, such as CsoR, CopY, and ArsR, prevent the transcription of genes encoding Cu-exporting P_1B_-type ATPases and Cu chaperones. On the other hand, periplasmic Cu concentrations are primarily sensed by two-component systems, such as CusRS or CopRS (22).

**FIG 1:**
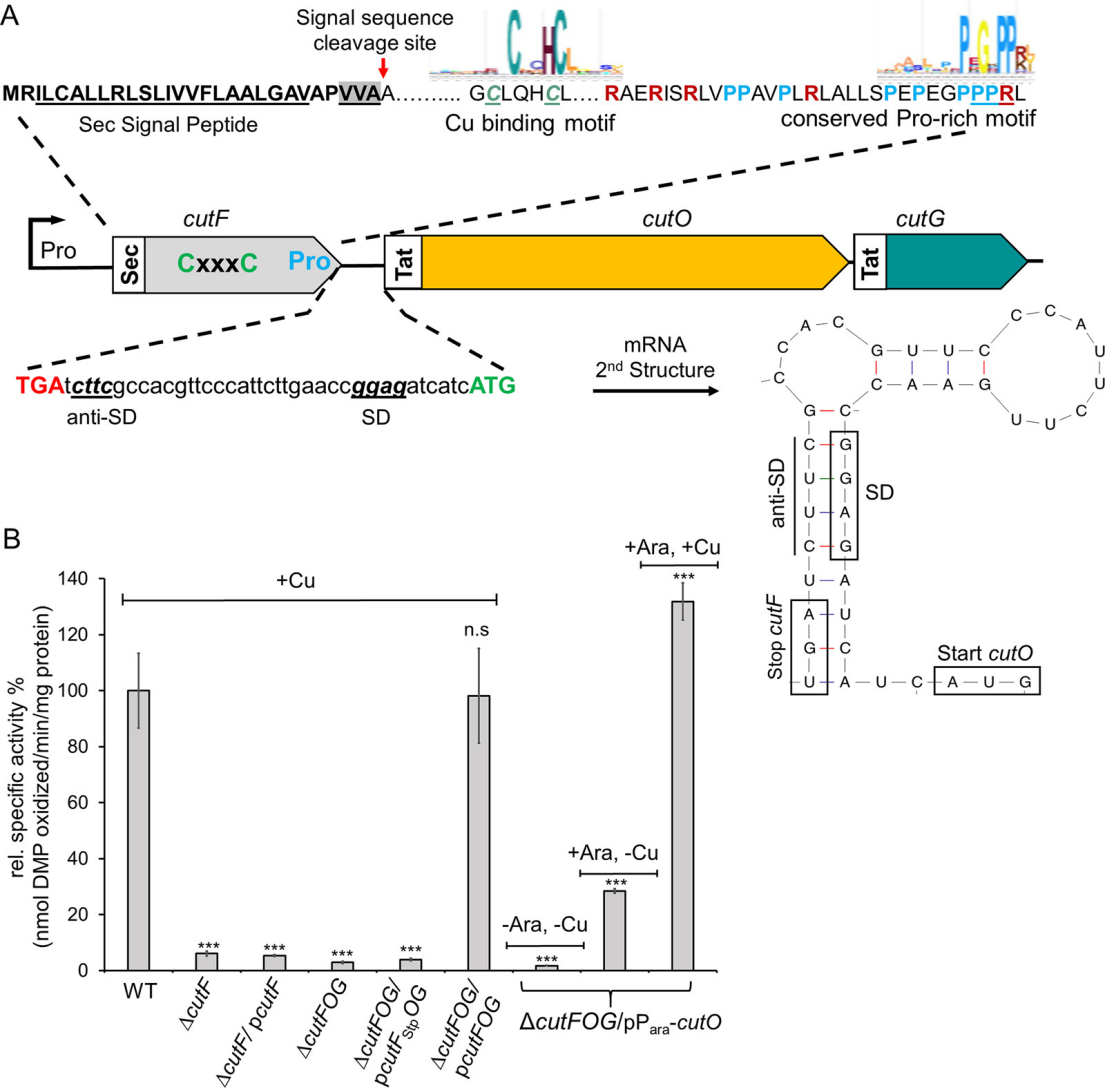
The structural integrity of the *cutFOG* operon is essential for CutO production. (A) Genetic organization of the *cutFOG* operon in R. capsulatus. The *cutFOG* operon encodes CutF, a predicted protein of unknown function, the multicopper oxidase CutO, and the copper chaperone CutG. CutF has a cleavable Sec signal sequence (Sec), and CutO and CutG contain a twin-arginine signal sequence (Tat). The putative Cu-binding motif (CxxxC) and the proline-rich C terminus of CutF are indicated. The intergenic *cutF-cutG* region forms a stem-loop structure that shields the Shine-Dalgarno sequence (SD) of *cutO*. (B) CutO activity of different R. capsulatus strains that lack either *cutF* (Δ*cutF*) or the entire *cutFOG* operon (Δ*cutFOG*). p*cutF* contains an N-terminally Flag-tagged copy of *cutF* under the control of its native promoter in the low-copy-number plasmid pRK415, and similarly, p*cutFOG* encodes epitope-tagged versions of the entire *cutFOG* operon with an N-terminal Flag-tag, a C-terminal Flag-tag, and a C-terminal Myc-His tag, respectively. The plasmid p*cutF*_Stp_*OG* is a derivative of p*cutFOG* with a substitution of the *cutF* start codon with a stop codon. The plasmid pP_ara_-*cutO* contains the C-terminally Flag-tagged CutO under arabinose promoter control. The CutO activities were determined via a 2,6-DMP assay, using the periplasmic fractions (50 μg total protein) of appropriate strains after their growth on a MPYE-enriched medium. Where indicated, the MPYE medium was supplemented with 10 μM CuSO_4_ and 0.5% arabinose. The activity of the wild-type (WT) was set to 100%, and the relative activities of the indicated strains were calculated. Three independent experiments, consisting of three technical replicates in each case, were performed, and the error bars reflect the standard deviation (*n* = 9). The statistical analyses were performed with the Satterthwaite-corrected, two-sided Student’s *t* test, using the activity of the WT as a reference. *, *P* ≤ 0.05; **, *P* ≤ 0.01; ***, *P* ≤ 0.001; n.s., not significant.

In addition to transcriptional control, some Cu-response proteins are post-transcriptionally regulated. One example is the proteolysis of CueR by the AAA^+^ proteases Lon, ClpXP, and ClpAP (23). Another example is the multicopper oxidase CutO of R. capsulatus (15, 24). In response to Cu, the production of CutO is regulated by a mRNA stem-loop (SL) that is located between *cutF* and *cutO* and strictly requires the presence of an intact *cutF* in the tricistronic *cutFOG* operon (Fig. 1A). Although *cutF* can be translated into protein *in vitro*, CutF is not detectable *in vivo* by either immune detection or mass spectrometry (15, 25). Moreover, the genetic complementation of a Δ*cutF* mutant is only possible when the *cutFOG* operon is kept intact, indicating that *cutF* must be located immediately upstream of *cutO* to execute its function. Bioinformatic searches identified CutF as a member of the DUF2946-like family of proteins, which are abundant in Pseudomonadota and are often encoded upstream of cuproenzymes, periplasmic Cu chaperones, and Cu transporters (15).

Despite their abundance, the functions of CutF-like proteins are unknown. Our findings show that the cotranslational export of the putative Cu-binding protein CutF is delayed by a Cu-induced, translational pausing-like process that causes the unfolding of a downstream mRNA SL, which allows for the translation of the adjacent *cutO*. Bioinformatic analyses reveal that such metabolic sensing mechanisms may represent a more common and largely unexplored regulatory principle in bacteria.

## RESULTS

### The structural integrity of the *cutFOG* operon and the translation of *cutF* are required for Cu-dependent CutO production.

CutF is essential for CutO production (15), with a Δ*cutF* strain showing the same low CutO activity as that of a Δ*cutFOG* strain (Fig. 1B). Whereas the Δ*cutFOG* strain is fully complemented by the ectopic expression of the *cutFOG* operon, the lack of CutO activity and the associated Cu sensitivity of a nonpolar Δ*cutF* mutant cannot be complemented in *trans* by a plasmid expressing solely *cutF* (p*cutF*) (Fig. 1B; Fig. S1A). This indicates that the *cutF* product can execute its function only when in *cis* to *cutO*. Since CutF is not detectable in cells (15, 25), whether *cutF* is translated *in vivo* was probed by replacing its predicted ATG start codon with a stop codon in a plasmid containing the intact *cutFOG* operon (p*cutF_Stp_OG*). This construct failed to restore CutO activity, whereas the same plasmid containing *cutF* with its start codon (p*cutFOG*) allowed for wild-type CutO activity and the associated Cu-tolerance (Fig. 1B; Fig. S1B). Thus, *cutF* translation *in vivo* is needed to support Cu-dependent CutO production.

10.1128/mbio.03040-22.1 FIG S1 Cu sensitivity phenotypes of different R. capsulatus strains. (A) Cu sensitivity phenotypes of the plasmid-encoded, N-terminally Flag-tagged CutF variant (p*cutF*) in the Δ*cutF* strain. Growth phenotypes were tested under the respiratory (Res) growth condition on MPYE medium supplemented with 400 μM CuSO_4_ or without CuSO_4_ at 35°C for 3 to 4 days. (B) The Cu sensitivity of the Δ*cutFOG*/p*cutF*_Stp_*OG* strain carrying the start to stop codon substitution of CutF was tested via spot assay. The strains were serially diluted (see Materials and Methods) and grown as described in panel A. (C) Immunoblot analysis of the periplasmic fractions (50 mg protein) of the Δcut*FOG* strain carrying pPara-*cutO* using α-Flag antibodies. As a control, the periplasmic fraction of the Δcut*FOG* strain carrying pcut*FOG* under the endogenous promoter was analyzed. A representative Western blot of 3 biological replicates is shown. The lower part of the SDS-PAGE was Coomassie stained and serves as a loading control. (D) The Cu sensitivity phenotypes of the SL mutated derivatives of the *cut* operon lacking *cutF.* The growth conditions were as described in panel A, and details on the stem-loop mutations are presented in Fig. S2. Download FIG S1, TIF file, 2.1 MB. Copyright © 2023 Öztürk et al. 2023 Öztürk et al. https://creativecommons.org/licenses/by/4.0/ This content is distributed under the terms of the Creative Commons Attribution 4.0 International license.

An analysis of the *cutFOG* transcript showed that *cutO* is preceded by a SL that shields the predicted *cutO* Shine-Dalgarno (SD) sequence (24) (Fig. 1A). The unfolding of this region is likely required for CutO synthesis. Whether CutF is required for SL unfolding was addressed via the ectopic expression of *cutO* under the control of an arabinose-controlled promoter in the Δ*cutFOG* strain in the absence of SL and CutF (pP_ara_-*cutO*). CutO activity was detectable upon the addition of arabinose, and this activity was increased by Cu supplementation (Fig. 1B). Immune-detection showed that Cu barely affects the steady-state amount of Flag-tagged CutO (Fig. S1C), indicating that the addition of Cu has no major effect on the CutO levels but rather enhances CutO enzymatic activity, which has been also observed before (15) Thus, CutO can be readily produced in the absence of both CutF and SL when expressed from a *cutO* gene in *trans*.

**FIG 2:**
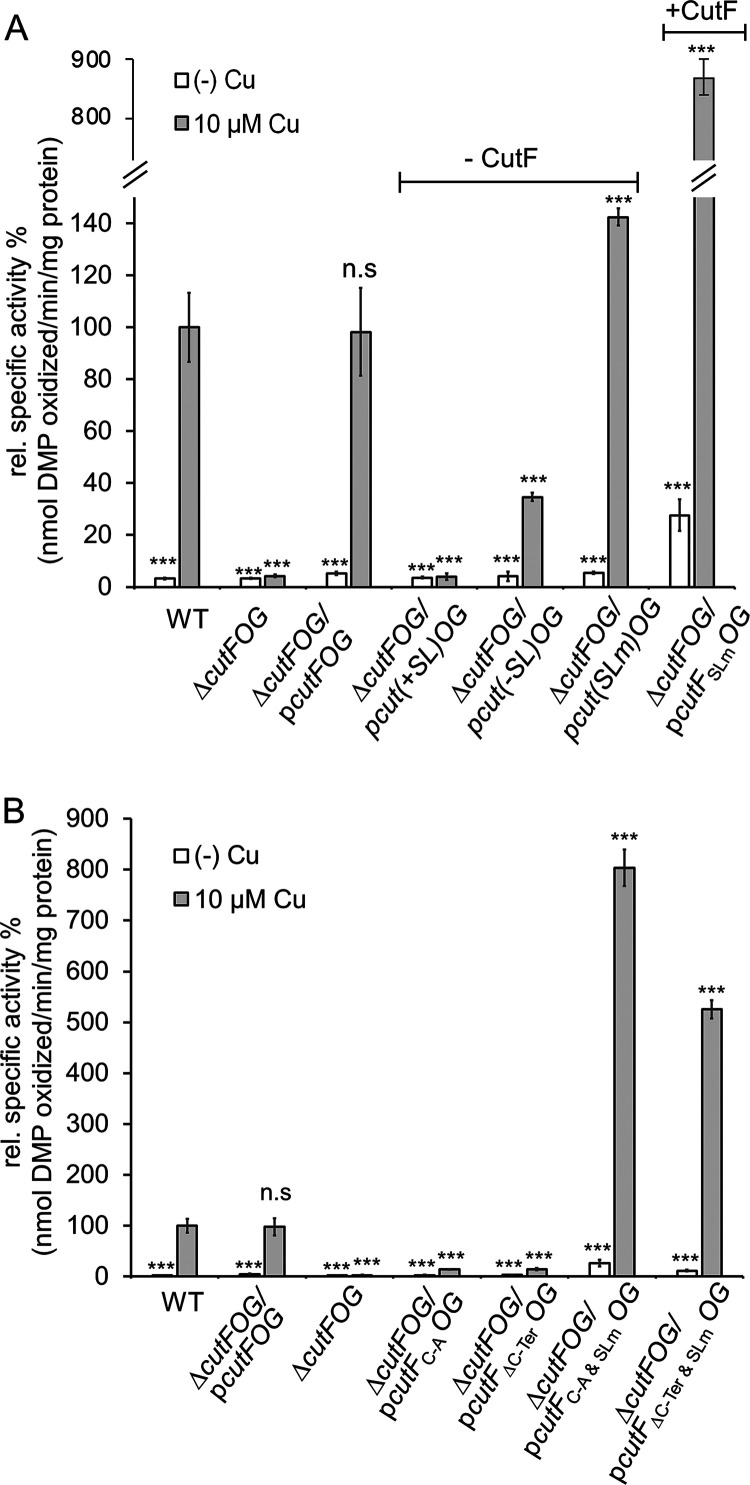
CutF is essential for Cu-induced CutO production in the presence of the stem-loop but not in its absence. (A) CutO activity was determined as described in Fig. 1. p*cutF(SLm)OG* is a derivative of p*cutFOG* in which the anti-SD within the SL is mutated. p*cut(+SL)OG* encodes just *cutOG* and contains the stem-loop upstream of *cutO*, whereas p*cut(−SL)OG* lacks the stem-loop and p*cut(SLm)OG* contains *cutOG* with a mutated anti-Shine-Dalgarno sequence (see Fig. S2A for details). The presence or absence of *cutF* is indicated on the top of the figure. (B) CutO activity of R. capsulatus Δ*cutFOG* cells expressing *cutF* with a mutated Cu-binding motif (C-A) or a truncated C terminus (ΔC-Ter) combined with an intact or mutated stem-loop (Slm). All of the mutations were located on the p*cutFOG* plasmid that carried the tagged versions of the genes. The CutO activities of the wild-type (WT), the Δ*cutFOG* strain, and the Δ*cutFOG* carrying the p*cutFOG* plasmid served as controls. The CutO activities of the indicated strains in panels A and B as well as the statistical analyses (*n* = 9) were calculated as before.

Whether CutF is required for the Cu- and SL-dependent production of CutO was tested using two constructs. Both lacked *cutF*, but only one contained SL (Fig. S2A). In the presence of SL and in the absence of *cutF* (p*cut(+SL)OG*), no significant CutO activity was observed, whereas in the absence of both SL and *cutF* (p*cut(−SL)OG*), some CutO activity (Fig. 2A) and the associated Cu-tolerance (Fig. S1D) were detectable. Considering that the SL could shield the SD sequence upstream of *cutO*, a third construct was tested, in which the anti-SD sequence was mutated (24) (Fig. S2A). The CutO activity in the Cu-supplemented cells containing this mutated SL (*SL_m_*) and lacking *cutF* (*cut(SLm)OG*) was higher than the activity observed in the absence of SL. This suggests that the elimination of the *cutF-SL* region also interferes with the stability of the *cutOG* mRNA, as seen earlier (15, 24). Further supporting this hypothesis, CutO activity increased almost 10-fold in cell extracts expressing *cutF* and *SLm* together (*cutF_SLm_OG*) (Fig. 2A), and immune detection showed that the CutO levels were strongly increased in cell extracts (Fig. S2B, *cutF_SLm_OG*). This showed that CutF is required for maximum Cu-dependent CutO production only in the presence of an intact *cutF-SL* region and not in the absence of the SL.

10.1128/mbio.03040-22.2 FIG S2 Construction of the SL mutated derivatives of the *cut* operon without *cutF.* (A) The entire *cutF*, excluding the last 4 aa sequence, was deleted from the p*cutFOG* plasmid to obtain the p*cut(+SL)OG* construct. In the p*cut(−SL)OG* plasmid, the entire *cutF* and anti-SD (a-SD) sequences were deleted. The *Cut(SLm)OG* construct was obtained by introducing the CTTC-AAAA substitution mutation (SLm) (underlined in red) on the a-SD sequence of the p*cut(+SL)OG* plasmid to prevent stem-loop formation. The SD and a-SD sequences are indicated by bold underlined letters. (B) Immunoblot analysis of the periplasmic fractions (100 mg total protein) of the indicated strains. α-Flag antibodies were used for the detection of CutO. A section of the corresponding SDS PAGE was stained with Ponceau-red and served as a loading control. Representative Western blots of three biological replicates are shown. (C) Immunoblot analysis using 100 mg of protein of the periplasmic fractions of appropriate cells related to *cutF_C-A_OG* and *cutF_DC-Ter_OG*. (D) α-Flag antibodies were used for the detection of CutO. Appropriate sections of the corresponding SDS PAGE or membrane blots were stained as a loading control with Ponceau-red or Coomassie, respectively. Representative Western blots of three biological replicates are shown. Download FIG S2, TIF file, 2.1 MB. Copyright © 2023 Öztürk et al. 2023 Öztürk et al. https://creativecommons.org/licenses/by/4.0/ This content is distributed under the terms of the Creative Commons Attribution 4.0 International license.

### Conserved motifs of CutF cooperate with SL for Cu-dependent CutO production.

CutF contains a putative Cu-binding motif (CxxxC) that is commonly seen in Cu-binding proteins as well as a conserved C-terminal proline-rich motif that is reminiscent of ribosomal stalling sequences (26, 27) (Fig. 1A). Both motifs are required for Cu-dependent CutO activity (15). Whether these motifs are important for increasing the accessibility of the *cutO* SD site was tested by using strains that expressed mutated CutF variants combined with *SLm*. When the cysteine residues of the Cu-binding motif were replaced by alanine (CutF_C-A_) in the presence of wild-type SL, no CutO activity was detectable. The same was also observed for a CutF variant lacking the proline-rich sequence (CutF_ΔC-Ter_). Conversely, both variants produced large amounts of Cu-dependent CutO activity when combined with *SLm* (Fig. 2B). Moreover, cells carrying either one of these double mutations (*cutF_C-A_* and *SLm* or *cutF_ΔC-Ter_* and *SLm*) produced much larger amounts of CutO than did wild-type cells or Δ*cutFOG* cells complemented with a plasmid carrying *cutFOG* in the absence or presence of Cu (Fig. S2C and D). Thus, both the Cu-binding and the proline-rich motifs of CutF cooperate with the SL for maximal Cu-dependent CutO production.

### The Sec signal peptide of CutF is required for Cu-dependent CutO production.

**FIG 3:**
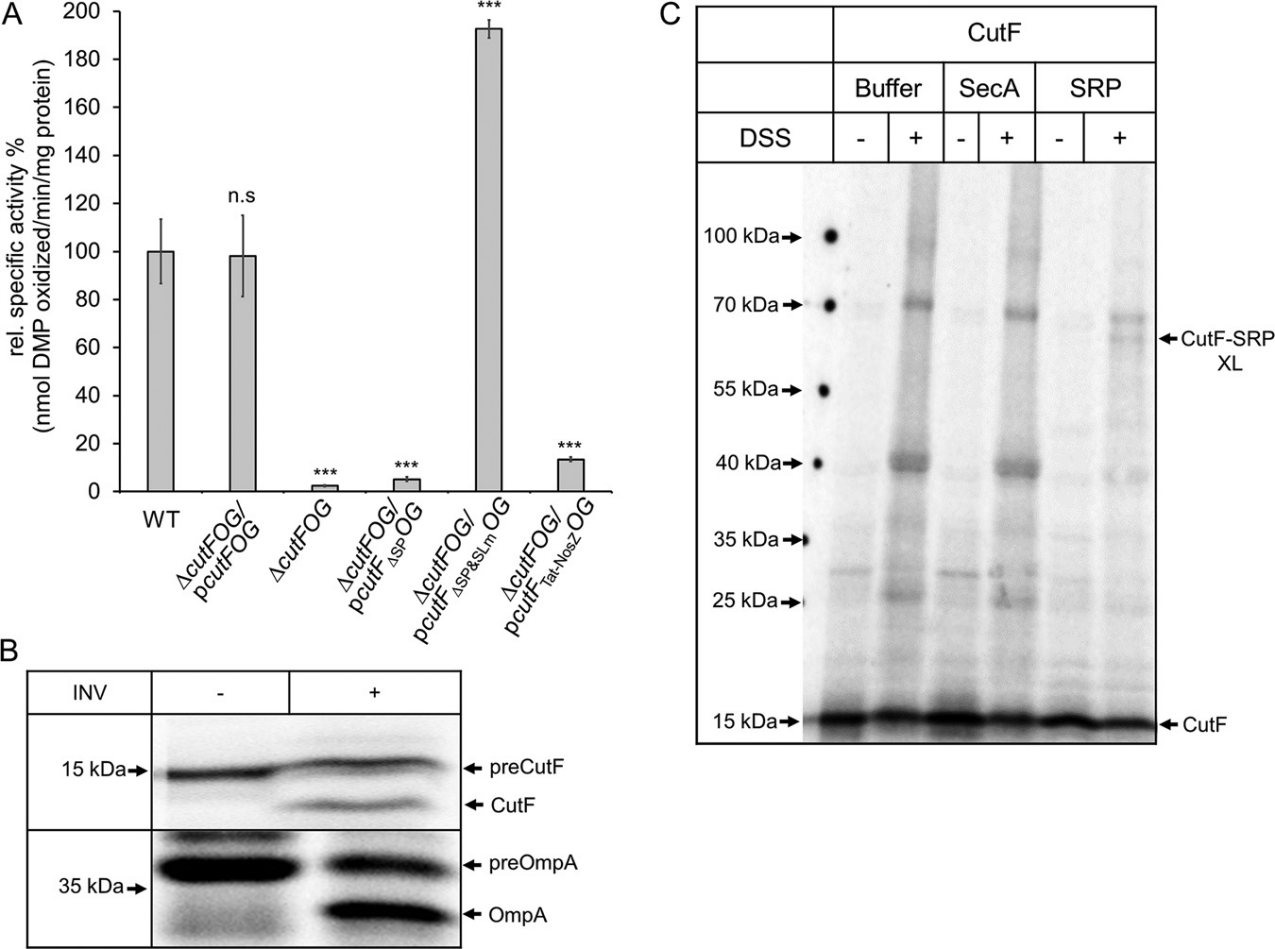
The Sec signal peptide is essential for CutF function. (A) CutO activity of R. capsulatus Δ*cutFOG* cells carrying p*cutFOG* variants with either a deletion of the *cutF* signal sequence (p*cutF_Δ_*_SP_*OG*) or a replacement of its Sec signal sequence with the NosZ twin-arginine signal sequence (p*cutF_Tat-NosZ_OG*). The deletion/replacement of the Sec signal sequence was also combined with the stem-loop mutation in the plasmid p*cutF*_ΔSP&SLm_*OG*. Cells were grown on MPYE medium supplemented with 10 μM CuSO_4_. The CutO activities of the indicated strains and the statistical analyses (*n* = 9) were calculated as before. (B) An E. coli transcription/translation system was employed for the *in vitro* synthesis of CutF in the absence and presence of E. coli inverted inner membrane vesicles (INV). The E. coli protein OmpA was used as a control. After *in vitro* synthesis, the radioactively labeled samples were separated via SDS-PAGE and were analyzed via phosphorimaging. Indicated are the signal sequence-containing precursors of CutF and OmpA (preCutF, preOmpA) and the mature CutF and OmpA proteins. (C) CutF was synthesized *in vitro* and incubated with either buffer or 36 ng/μL of purified SecA or signal recognition particle (SRP). The samples were subsequently treated with disuccinimidyl suberate (DSS) or buffer, separated via SDS-PAGE, and analyzed via phosphorimaging. Indicated are the *in vitro* synthesized CutF and the CutF-SRP cross-linking product (CutF-SRP XL).

A striking feature of the proteins encoded by *cutFOG* is that CutF has a predicted Sec signal peptide, whereas CutO and CutG contain Tat signal peptides that are typical for proteins translocated across the membrane in a folded or partially folded state (28). As the folding of Tat substrates occurs after their release from the ribosome, the Tat-system transports proteins post-translationally (29). In contrast, the Sec-system can act both post-translationally and cotranslationally (30, 31). Consequently, the cotranslational export of CutF might control the translation of the downstream encoded *cutO*. In this case, its Sec signal peptide should be essential for controlling CutO production by coupling the export of CutF to *cutO* translation. Indeed, when the signal peptide of CutF was deleted (*cutF*_ΔSP_*OG*), the activity and steady-state levels of CutO were decreased to the background levels of the Δ*cutFOG* strain (Fig. 3A; Fig. S3A). However, the signal peptide of CutF was dispensable when the SL was mutated (Fig. 3A; Fig. S3B). Thus, CutF export to the periplasm is required for CutO production only in the presence of the SL. Whether CutF export occurs via the SecYEG translocon was tested by replacing the Sec signal peptide of CutF with the Tat signal peptide of NosZ. This replacement resulted in a drastic decrease of CutO activity (Fig. 3A) and in reduced levels of CutO (Fig. S3A). Thus, seemingly, the translocation of CutF into the periplasm *per se* is insufficient, and CutF requires its Sec signal peptide to support full CutO production.

10.1128/mbio.03040-22.3 FIG S3 Immunoblot analysis of the periplasmic fractions (100 mg protein) of R. capsulatus strains. (A) Δ*cutFOG* cells carrying the p*cutFOG* variants p*cutF_Δ_*_SP_*OG* and p*cutF_Tat-NosZ_OG*. (B) Δ*cutFOG* cells carrying the (p*cutF_Δ_*_SP_*OG*) and p*cutF*_ΔSP&SL_*OG*. α-Flag antibodies were used for detecting CutO. A section of the corresponding SDS PAGE was stained with Coomassie and served as a loading control. Representative Western blots of three biological replicates are shown. In panels A and B, nonrelevant parts of the blot were cut out, and the two parts were reassembled, as indicated by the vertical lines. Download FIG S3, TIF file, 2.1 MB. Copyright © 2023 Öztürk et al. 2023 Öztürk et al. https://creativecommons.org/licenses/by/4.0/ This content is distributed under the terms of the Creative Commons Attribution 4.0 International license.

Although CutF has yet to be detected *in vivo*, its translation proficiency was confirmed using an *in vitro* transcription-translation system of E. coli (15). This system was used to determine whether the Sec signal sequence of CutF was cleaved in the presence of E. coli inner membrane vesicles (INVs) to obtain further evidence of its translocation by the SecYEG translocon. When CutF was synthesized *in vitro* in the absence of INVs, a single, radioactively labeled band corresponding to the expected size of CutF was seen (Fig. 3B). In the presence of INVs, a second band of lower molecular weight appeared, corresponding to mature CutF that lacked its signal peptide. As a control, signal sequence cleavage was monitored for OmpA, which is a model substrate used for protein translocation and signal peptide cleavage in E. coli (32) (Fig. 3B). Thus, CutF has a cleavable Sec signal peptide and can be translocated by the SecYEG translocon.

### The cotranslational export of CutF and the role of its C-terminal proline-rich motif.

The targeting of proteins containing Sec signal peptides to the SecYEG translocon can occur via either SecA or the signal recognition particle (SRP). SecA preferentially directs secretory proteins post-translationally to the SecYEG translocon, whereas SRP primarily targets membrane proteins cotranslationally (30). Whether CutF interacts with SecA or SRP was probed using an *in vitro* cross-linking approach. *In vitro* synthesized CutF was incubated with purified SecA or SRP, and this was followed by chemical cross-linking using disuccinimidyl suberate (DSS) (Fig. 3C). In the presence of SRP, the addition of DSS resulted in a radioactively labeled band at 65 kDa that corresponded in size to a cross-link between SRP and CutF. No specific cross-linking product was detected in the presence of SecA. Although the data do not exclude that CutF might also interact with SecA, they do show that SRP can target CutF to SecYEG and can support the cotranslational export of CutF.

**FIG 4:**
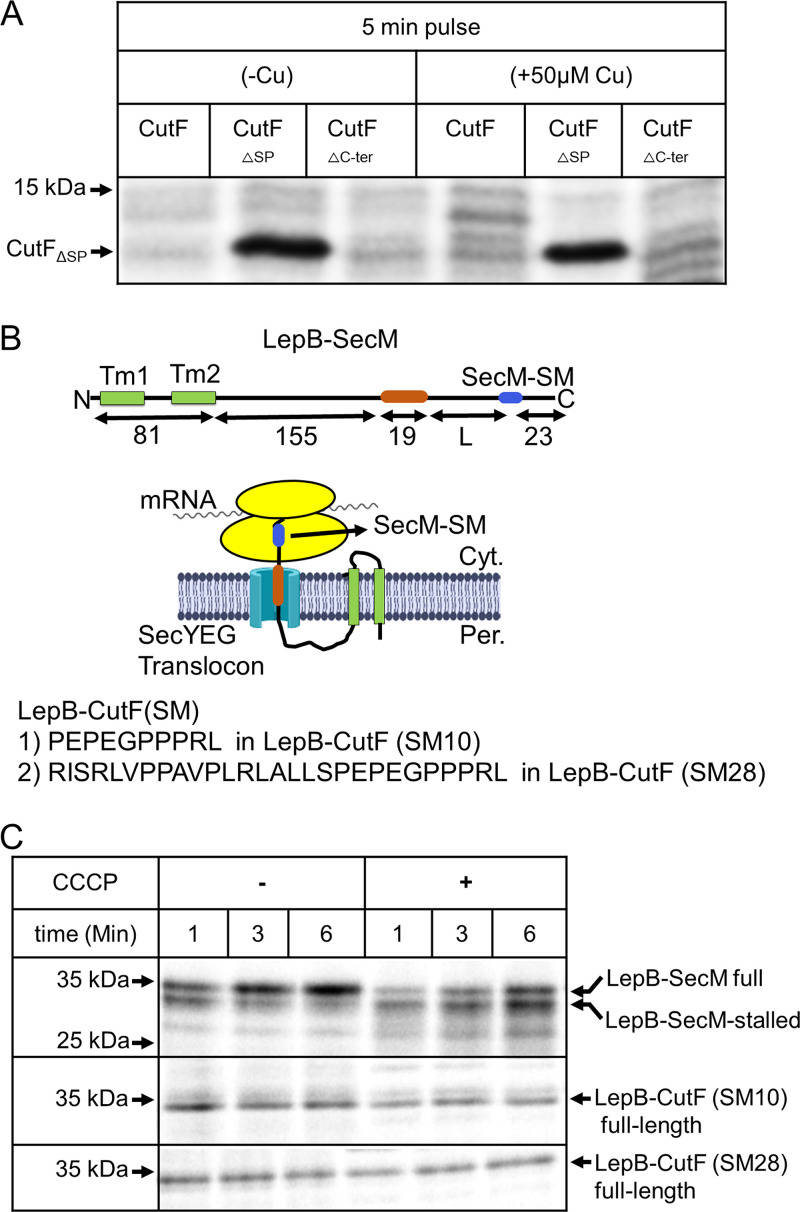
Secreted CutF is rapidly degraded. (A) CutF and its variants lacking either the signal sequence (CutF_ΔSP_) or the C-terminal proline-rich motif (CutF_ΔC-ter_) were expressed *in vivo* under *T7*-promoter control in E. coli cells and were labeled with [^35^S] methionine/cysteine. After 5 and 10 min of labeling, the whole cells were precipitated with trichloroacetic acid (TCA), separated via SDS-PAGE, and analyzed via phosphorimaging. Where indicated, labeling was performed in the presence of 50 μM Cu. The complete phosphorimaging covering 10 min of labeling is shown in Fig. S4A. (B) Cartoon showing the features of the translational stalling sensor LepB-SecM, which contains the first two transmembrane domains of LepB fused to a hydrophobic stretch (H) as well as the SecM stalling motif (SecM-SM) (upper panel). Sequences of the CutF stalling motifs that were used to replace the SecM-SM in the LepB stalling sensor, resulting in LepB-CutF(SM10) and LepB-CutF(SM28) (lower panel). (C) LepB-SecM, LepB-CutF(SM10), and LepB-CutF(SM28) were expressed *in vivo*, radiolabeled, and processed as described in panel A. Where indicated, the protonophore CCCP was added. Indicated are the full-length LepB-SecM (upper band), its stalled version (lower band), and the full-length versions of LepB-CutF(SM10) and LepB-CutF(SM28).

The C terminus of CutF contains a conserved, proline-rich sequence that is required for Cu-dependent CutO production and is reminiscent of ribosomal stalling sequences (Fig. 1A and 2B) (15). Cis-acting translational modulators containing C-terminal ribosomal stalling sequences frequently execute the unfolding of SLs that cover the SD sequences of downstream genes (33–35). To probe whether the proline-rich motif of CutF acts as a ribosomal stalling sequence, *in vivo* metabolic labeling experiments were performed in E. coli. CutF was expressed under *T7*-promoter control, endogenous transcription was blocked by rifampicin, and cells were supplemented with ^35^S-labeled methionine/cysteine. The data show that CutF is not detectable either in the absence or presence of Cu unless its signal sequence is deleted (CutF_ΔSP_) (Fig. 4A; Fig. S4A). This indicates that the cytoplasmic form of CutF is stable but that it is rapidly degraded upon translocation into the periplasm. This observation is consistent with the plausible role of CutF serving as a transient sensor to monitor the periplasmic Cu content. However, as neither CutF nor its variant lacking the proline-rich motif (CutF_ΔC-ter_) was detectable, it remains unanswered whether its C terminus contains a ribosomal stalling sequence.

10.1128/mbio.03040-22.4 FIG S4 Expression of CutF, CutF_ΔSP_, and CutF_ΔC-ter_ *in vivo* under *T7*-promoter control in E. coli cells. (A) After 5 and 10 min of labelling with [^35^S] methionine/cysteine, whole cells were precipitated with trichloroacetic acid (TCA), separated via SDS-PAGE, and analyzed via phosphorimaging. (B) Pulse-labeling of LepB-SecM in the presence and absence of Cu. The experiment was performed as described in Fig. 4, and the full-length LepB–SecM (upper band) and its stalled version (lower band) are indicated. Download FIG S4, TIF file, 2.1 MB. Copyright © 2023 Öztürk et al. 2023 Öztürk et al. https://creativecommons.org/licenses/by/4.0/ This content is distributed under the terms of the Creative Commons Attribution 4.0 International license.

Many ribosomal stalling sequences have been identified and characterized via fusion to upstream sequences, such as the leader peptidase LepB (36, 37) (Fig. 4B). Indeed, the *in vivo* pulse-labeling of LepB fused to SecM (LepB-SecM) with its intact ribosomal stalling motif (SecM-SM) results in two bands, reflecting the full-length and stalled forms (Fig. 4C). Other studies have shown that the formation of a stalled LepB-SecM band requires an intact SecM stalling motif (36). When the SecM-SM motif of LepB-SecM is replaced with the C-terminal 10 (SM10) or 28 (SM28) amino acids of CutF, encompassing its proline-rich motif and resulting in LepB-CutF (SM10) or (SM28) (Fig. 4B), only the full-length proteins, not the stalled forms, were detectable (Fig. 4C).

Over time, the arrested LepB-SecM is converted to its full-length, demonstrating that protein translocation across the membrane provides sufficient force to extract the stalling peptide out of the ribosome (36, 37). The protonophore carbonyl cyanide m-chlorophenyl hydrazine (CCCP) inhibits protein translocation (36, 37) and reduces the conversion of the stalled LepB-SecM into full-length (Fig. 4C). Similar analyses with LepB-CutF show that the addition of CCCP slightly reduced its production, but no corresponding stalled form was observed (Fig. 4C). Thus, the proline-rich sequence of CutF does not act, *per se*, as a strong stalling sequence when tested using LepB-SecM. However, as these constructs lacked the Cu-binding motif, none of the effects of Cu could be tested.

### Cu addition decreases the cotranslational export of CutF.

**FIG 5:**
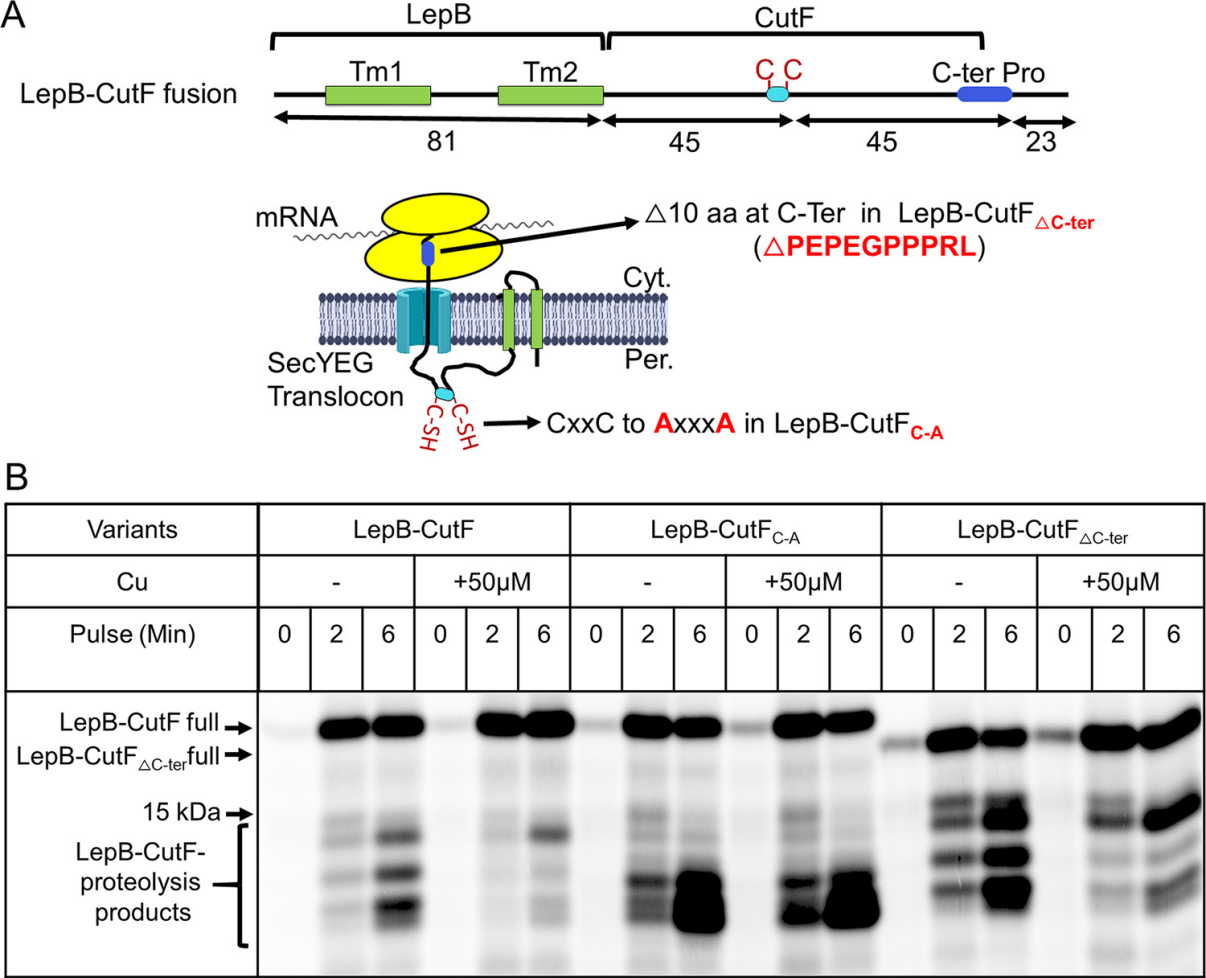
Cu enhances translational stalling and reduces the proteolysis of LepB-CutF fusion. (A) Cartoon representation of the LepB-CutF fusion and its variants that lack either the Cu-binding motif (LepB-CutF_C-A_) or the C-terminal proline-rich motif (LepB-CutF_ΔCter_). The mature part of CutF and of both variants were immediately fused to LepB after the second TM helix. (B) Pulse-labeling of LepB-CutF and both variants, as described in Fig. 4, in the presence or absence of 50 μM CuSO_4_. After the 0-, 2-, and 6-min pulses, the samples were precipitated and analyzed via phosphorimaging. Indicated are the full-length LepB-CutF and the proteolysis fragments.

As Cu-dependent CutO production requires both the Cu-binding and the proline-rich motifs of CutF (Fig. 2B) (15), the mature part of CutF (90 amino acids) was fused to the first two transmembrane domains of LepB. Further, a 23 amino acid-long peptide was added to the C terminus after the proline-rich motif, thereby generating a 194 residue-long LepB-CutF fusion to detect its possible arrested form (Fig. 5A). Pulse-labeling resulted in an approximately 21 kDa product, which corresponds to the full-length LepB-CutF being the dominant species. However, the arrested LepB-CutF, which should migrate around 19 kDa, was not detected (Fig. 5B, left panel). Instead, several bands of approximately 12 to 14 kDa were seen, likely corresponding to the proteolytic cleavage products of the secreted CutF portion of LepB-CutF. The proteolytic sensitivity of secreted CutF (Fig. 4A) as well as the stability of the LepB-SecM (36) and LepB-CutF(SM) fusions (Fig. 4C) have been seen before.

Provided that this proteolysis is indeed associated with the export of CutF into the periplasm, we reasoned that conditions that enhance its translational stalling and consequently decrease its export should reduce proteolysis. When pulse-labeling was repeated in the presence of Cu (50 μM), only full-length LepB-CutF and its 14 kDa fragment were visible. The other proteolytic cleavage products were largely absent (Fig. 5B, left panel), suggesting that the addition of Cu enhanced the translational stalling of LepB-CutF. However, it was recently shown that Cu can also inhibit protein export via the Sec61 translocon (38). To exclude the possibility of the Cu-induced inhibition of the homologous SecYEG translocon, the LepB-SecM fusion was used as a control (Fig. S4B). The addition of Cu did not inhibit the translocation of LepB-SecM, confirming that the reduction of LepB-CutF proteolysis does not arise from the inhibition of the SecYEG translocon. Thus, the Cu-induced reduction of LepB-CutF proteolysis suggests its decreased export, possibly via a ribosomal stalling-like process.

This Cu-dependent process was further investigated using the LepB-CutF variants lacking either the Cu-binding or the proline-rich motifs (LepB-CutF_C-A_ or LepB-CutF_ΔC-ter_) (Fig. 5A). When the pulse-labeling experiment was repeated with LepB-CutF_C-A_ or LepB-CutF_ΔC-ter_ (Fig. 5B, middle and right panels, respectively) the addition of Cu at best slightly decreased proteolysis, unlike the wild-type LepB-CutF case. Thus, the occurrence of this ribosomal stalling-like process requires the presence of both the Cu-binding and the proline-rich motifs of CutF. The identity of the LepB-CutF proteolysis products and the basis of the slightly different proteolysis patterns seen with different LepB-CutF variants remain unknown. A possibility is that they might arise from their slightly different structures and interactions with Cu, but this needs to be further explored.

### CutF-like proteins are clustered in several sequence similarity network clusters.

**FIG 6:**
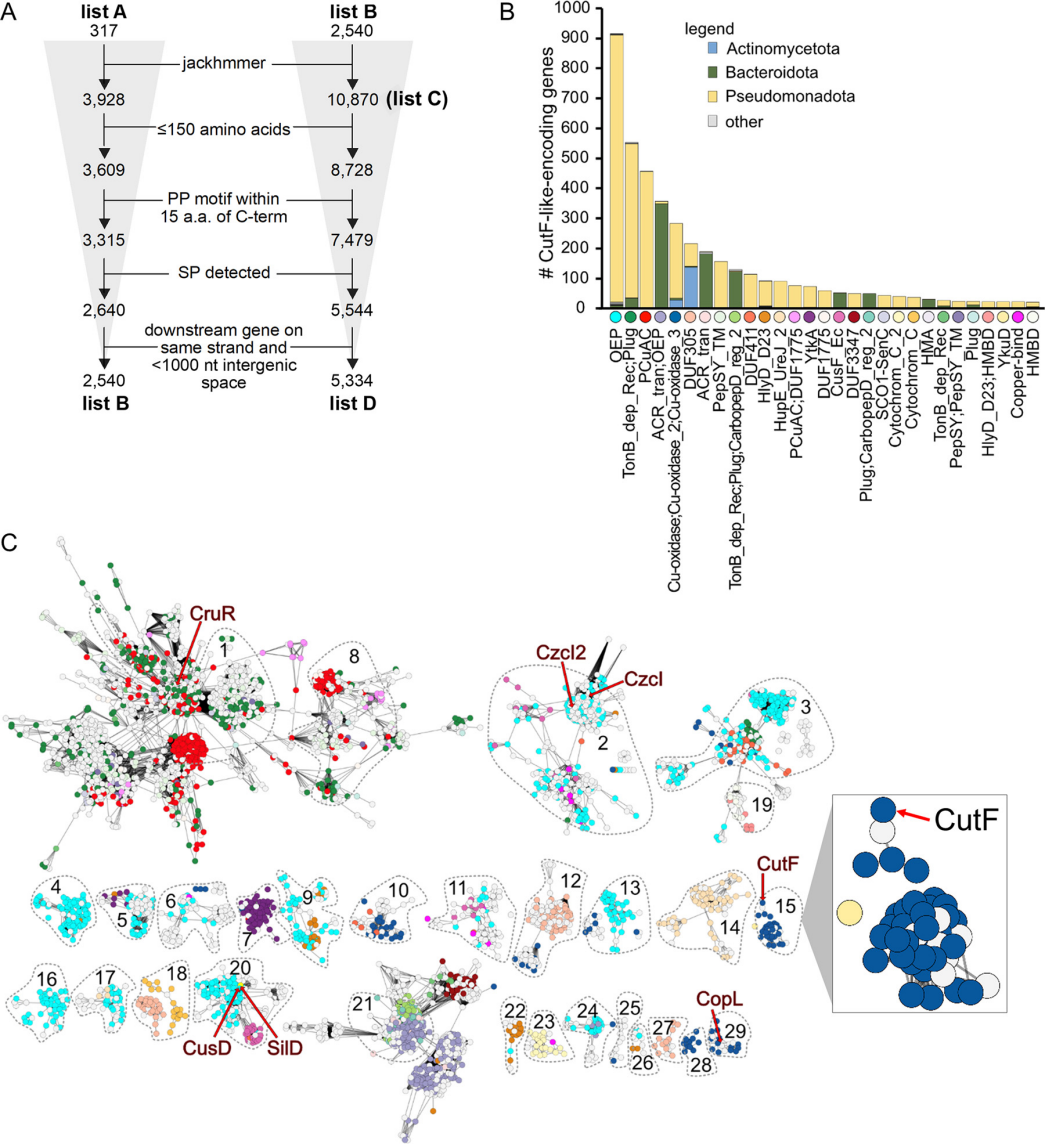
Identification and cluster analyses of CutF-like proteins. (A) Analysis workflow for the identification of the CutF-like proteins used in the downstream analyses. List A is from our previous studies (15). Lists B, C, and D were generated in this study. (B) The types of next-door neighbors identified downstream of the CutF-like genes from list D. The Pfam domain description for each class of neighbor is shown. Only neighbor classes with at least 20 instances (i.e., in 20 separate genomes) are shown. Phyla other than Pseudomonadota, Actinomycetota, and Bacteroidota are minor (i.e., no more than 5 examples for any given category; gray bars represent these minor “other” phyla). (C) The network containing proteins from list D (alignment score 5, nodes collapsed based on 100% similarity). The list B protein clusters are outlined and numbered. Nodes not connected to one of these clusters are not shown but can be found in Fig. S4. The nodes representing CutF, CruR, CopL, SilD, and CusD are labeled. Each node (each circle in the network) is colored based on its downstream neighbor, according to panel B. The CutF cluster is duplicated and enlarged on the right.

Our earlier finding that CutF-like proteins are widespread in Pseudomonadota (15), combined with the insights that emerged during this work, enticed us to investigate the conservation of the sensing mechanisms by CutF-like proteins as well as the identities of the proteins that they may regulate. A large-scale bioinformatic approach analyzing available protein sequences, their genomic contexts, and the putative intergenic RNA motifs found downstream of *cutF-*like genes was used. Starting with our previous rule-based list (list A, 317 entries) (Fig. 6A) of CutF-like proteins encoded by genes neighboring *cutO* (15), an iterative search using jackhammer against the Reference Proteomes in the UniProt database was carried out. Unlike previously (15), the analysis was not limited to Pseudomonodota, and sequences with matches to annotated domains were not removed. Several filters were applied for sequence characteristics at the protein (<150 amino acids long, presence of a proline-rich [PP] motif within the 15 C-terminal residues, and an N-terminal putative signal peptide [SP]), and at the intergenic (downstream gene on the same strand with an intergenic space of <1,000 nucleotides) levels (see Materials and Methods for details) (Fig. 6A). This yielded an initial list (list B) of 2,540 proteins that can be clustered into 29 sequence similarity network (SSN) clusters (1 to 29, each with >10 members) (Fig. 6C; Fig. S5). The hidden Markov model (HMM)-profile that was generated for each of the 29 SSN clusters showed distinct putative metal-binding sites with cysteines, histidines, or a combination of both (Fig. S6). In particular, the SSN clusters 14 and 17 are comprised of CutF-like proteins with only histidine-rich motifs (Fig. S6).

10.1128/mbio.03040-22.5 FIG S5 Analysis of the network containing proteins from list B (alignment score 5, nodes collapsed based on 100% similarity). (A) Protein clusters containing 10 or more members are colored and labeled. Nodes representing CutF, CopL, SilD, and CusD are labeled. The triangles with red outlines are from list A. (B) The nodes are colored based on the downstream gene. Download FIG S5, TIF file, 2.1 MB. Copyright © 2023 Öztürk et al. 2023 Öztürk et al. https://creativecommons.org/licenses/by/4.0/ This content is distributed under the terms of the Creative Commons Attribution 4.0 International license.

10.1128/mbio.03040-22.6 FIG S6 Sequence logos representing the profile-HMM generated using list B clusters. The number to the right refers to the cluster number in Fig. 6C. The “CutF” logo represents the profile-HMM from a search using CutF against UniProt with phmmer followed by jackhammer for 5 iterations. Download FIG S6, PDF file, 1.6 MB. Copyright © 2023 Öztürk et al. 2023 Öztürk et al. https://creativecommons.org/licenses/by/4.0/ This content is distributed under the terms of the Creative Commons Attribution 4.0 International license.

To increase the detection of CutF-like proteins, these 29 SSN clusters were individually searched using jackhammer against the Reference Proteomes in the UniProt database. The results were combined in a list (list C, 10,870 entries), and the same filters that were used for List B were applied (Fig. 6A). The final list (list D) contains 5,334 CutF-like proteins from 2,804 unique UniProt proteomes. These proteins are unequally distributed among many phyla, with most of them being from Pseudomonadota (55%) and Bacteriodota (40%) (normalizing to the number of available proteomes with at least one CutF-like protein in each case; otherwise, 76% and 19%, respectively), whereas those from Chloroflexota and Spirochaetota are scarce (Fig. S7A).

10.1128/mbio.03040-22.7 FIG S7 Phyla containing a CutF-like protein (A). Distribution of intergenic lengths between genes encoding CutF-like proteins and the downstream gene from list D (B). Presence of signal sequences in CutF-like proteins and the downstream encoded proteins (C) (shown as pairs). SP, Signal peptide (Sec/SPI); LIPO, lipoprotein signal peptide (Sec/SPII); TAT, TAT signal peptide (Tat/SPI); TATLIPO, TAT lipoprotein signal peptide (Tat/SPII); PILIN, pilin-like signal peptide (Sec/SPIII); Other, no signal peptide detected. Download FIG S7, TIF file, 2.1 MB. Copyright © 2023 Öztürk et al. 2023 Öztürk et al. https://creativecommons.org/licenses/by/4.0/ This content is distributed under the terms of the Creative Commons Attribution 4.0 International license.

### The genomic contexts of CutF-like proteins indicate that not all are Cu-specific, nor are they isofunctional.

Genomic context analyses indicate that most CutF-like proteins with cysteine-rich motifs (i.e., all except for the SSN clusters 14 and 17) are located upstream of genes involved in Cu homeostasis (Fig. 6B) (File S1). These include outer membrane efflux proteins (OEP) associated with RND efflux systems (OEP; InterPro: PF02321; often annotated as CusC/CzcC/SilC), acriflavine resistance proteins (ACR_tran; InterPro: PF00873; often annotated as CusA/CzcA/SilA), CusB/CzcB (HlyD_D23; InterPro: PF16576) and CusF (InterPro: PF11604) (Fig. 6B). After OEP, the next most frequent neighbors of the CutF-like proteins are the TonB-like proteins and the PCu_A_C-like periplasmic Cu chaperones. Strikingly, the neighboring genes to the CutF-like proteins with only histidine-rich motifs (SSN clusters 14 and 17, Fig. S6) belong to the HupE/UreJ family of proteins, which are often implicated in nickel and cobalt homeostasis. Thus, the overall findings suggest that not all CutF-like proteins are Cu-specific and that some might bind other metal ions, such as nickel and cobalt.

List D contains several previously studied proteins in addition to R. capsulatus CutF, which is located in the SSN cluster 15. For example, CruR from Bordetella pertussis (encoded by *bp2923*), located in SSN cluster 1 (Fig. 6C), was recently identified as an upstream ORF (uORF) that post-transcriptionally regulates the production of a TonB-like transporter. However, in the presence of Cu, CruR was suggested to relieve ribosomal stalling (39), unlike CutF, which enhances a similar process. Thus, CutF and CruR do not appear to be isofunctional. Further, CopL from Stenotrophomonas maltophilia (encoded by *smlt2449*), located in SSN cluster 29, is the most similar protein in UniProt to CopL from Xanthomonas perforans*. Xanthomonas copL* is required for the Cu-regulated expression of the downstream copper oxidase CopA, and, like *cutF*, *copL* does not act in *trans*, suggesting that CutF and CopL may be isofunctional (40).

### The modes of action of different CutF-like proteins are seemingly different.

**FIG 7:**
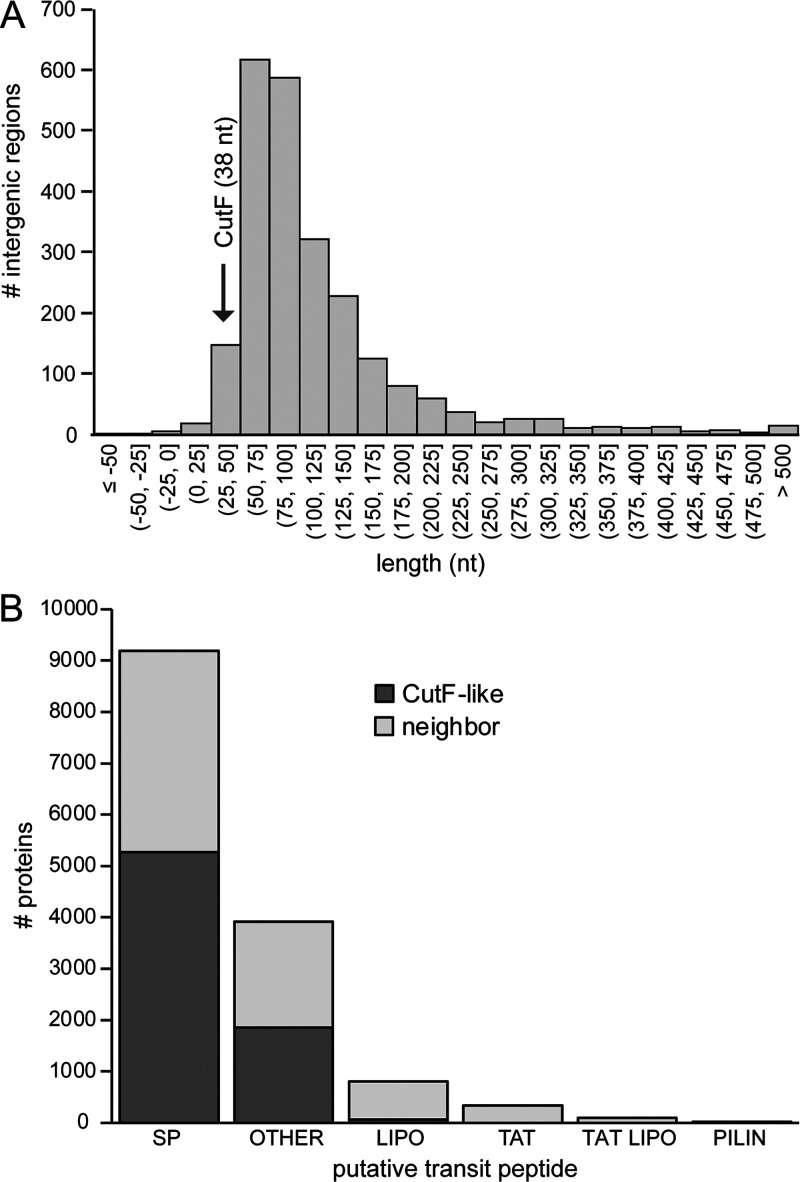
Features associated with CutF-like genes/proteins and neighbors. (A) Distribution of intergenic lengths between genes encoding CutF-like proteins and the downstream gene from list B. (B) Presence of signal sequences in CutF-like proteins and in the downstream encoded proteins, shown individually. SP, signal peptide (Sec/SPI); LIPO, lipoprotein signal peptide (Sec/SPII); TAT, TAT signal peptide (Tat/SPI); TATLIPO, TAT lipoprotein signal peptide (Tat/SPII); PILIN, pilin-like signal peptide (Sec/SPIII); Other, no signal peptide detected.

Provided that R. capsulatus CutF achieves Cu-dependent CutO production by unfolding a downstream SL via a translational stalling-like mechanism, we inquired whether this process is also employed by other CutF-like proteins for the regulation of the translation of their neighboring genes. We determined the length of the intergenic regions between the *cutF*-like genes and their downstream genes, as SL unfolding via translational stalling on upstream proteins is likely limited to short intergenic regions. The determination of the lengths of the intergenic regions downstream of the CutF-like genes in list B and list D shows that most consist of fewer than 100 nucleotides (Fig. 7A; Fig. S7B). Remarkably, some CutF-like proteins have short intergenic regions (<10 nucleotides) or even overlap their downstream neighbors. Nearly 60% of these cases encode either the cuproenzyme nitrous-oxide reductase or its transcriptional regulator NosR/NirR (41). This suggests that other type(s) of cotranslational Cu sensing mechanism(s), distinct from that seen with R. capsulatus CutF, might regulate the production of nitrous oxide reductase. Further, although most CutF-like proteins contain putative Sec signal sequences, such as R. capsulatus CutF (Fig. 7B), not all downstream proteins have a Tat signal sequence as does CutO. Indeed, the downstream proteins frequently contain Sec and other signal sequences (Fig. 7B; Fig. S7C), further suggesting that not all CutF-like proteins employ identical modes of regulation.

## DISCUSSION

CutF belongs to the abundant DUF2946 protein family, and our bioinformatic analyses show that they are frequently found upstream of genes that encode proteins that are involved in heavy metal detoxification. Most CutF-like proteins contain a cleavable Sec signal sequence, a Cu-binding motif in the mature part, and a C-terminal proline-rich sequence that is reminiscent of translational stalling sequences. The presence of an intact *cutF* located immediately upstream of *cutO* is essential for the Cu-dependent production of the multicopper oxidase CutO, even though CutF was not detected in whole cells. This may suggest that *cutF* produces a regulatory RNA that controls CutO production. However, our data demonstrate that the translation of *cutF* is required for CutO production and that this is followed by rapid CutF proteolysis. (i) *In vitro* transcription/translation experiments confirm the production of CutF and the cleavage of its signal sequence in the presence of membranes. (ii) In cells, replacing the initiator ATG codon of *cutF* with a TAG stop codon prevents CutO production. (iii) *In vivo* pulse-experiments show the cytoplasmic accumulation of CutF in the absence of its signal sequence, indicating that proteolysis occurs after its export into the periplasm. (iv) CutF supports full CutO production only when secreted into the periplasm via a Sec signal sequence, not when this Sec signal sequence is deleted or even replaced by a Tat signal sequence. Moreover, Cu-dependent CutO production also requires the presence of an intact Cu-binding motif in CutF.

Our data further demonstrate that the function of CutF is invariably linked to the presence of the SL between *cutF* and *CutO*. Previous data had indicated that this SL might shield the Shine-Dalgarno sequence of *cutO* and requires unfolding to allow for *cutO* translation (24). Our data now show that the translation and the cotranslational export of CutF are both required for this unfolding event, in conjunction with the presence of intact Cu-binding- and proline-rich motifs. Taken together, these observations support the role of CutF as a periplasmic Cu sensor that regulates *cutO* translation in response to extracytoplasmic Cu availability.

Proline-rich motifs often act as ribosomal stalling sequences and slow down translation due to inefficient peptide-bond formation at the ribosome (26, 27). The presence of positively charged residues in close proximity to the proline-rich motif, as observed for CutF (Fig. 1A), further reduces translational speed (42). The C-terminal stalling sequences that regulate the translation initiation of downstream genes have been observed with several proteins. Examples include the secreted proteins SecM, VemP, and MifM, which regulate the production of SecA, SecDF, and YidC2, respectively, in response to the cellular protein export status (33–35, 43). The release of SecM-, VemP-, or MifM-induced translational stalling depends on the SecYEG translocon, which provides sufficient force to extract the stalling sequence out of the ribosome. When SecYEG activity declines, translational stalling leads to the unfolding of a SL in the intergenic regions and increases the translation initiation of the downstream ORFs on the same mRNA (33–35, 43). The SL unfolding is likely achieved by the RNA helicase activity of the ribosome, which can unwind RNA secondary structures that are in its proximity (44).

**FIG 8:**
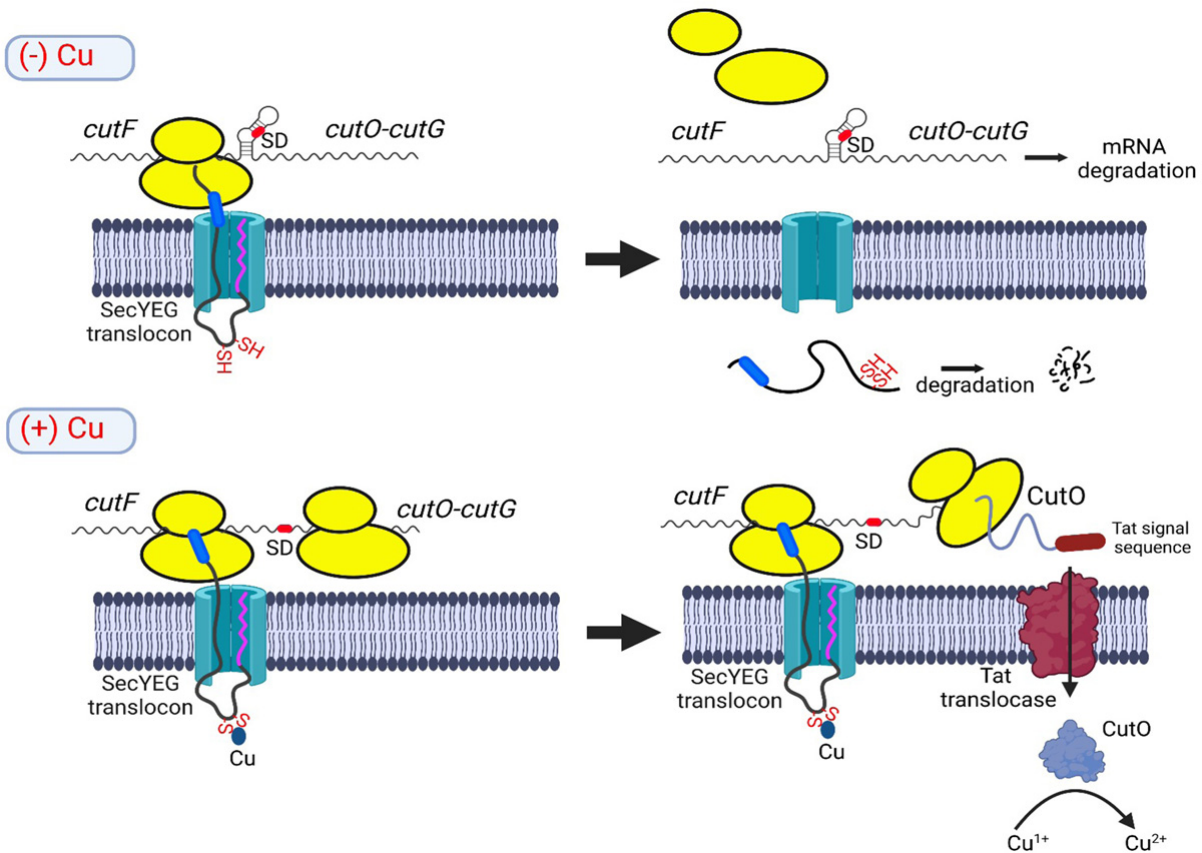
Model for Cu-controlled CutO production via the periplasmic Cu sensor CutF. CutF contains a cleavable signal sequence (magenta) and is cotranslationally targeted to the SecYEG translocon by SRP. In the absence of Cu (upper panel), the C-terminal stalling sequence (blue) is pulled out of the ribosome by the translocation activity of the SecYEG translocon, and CutF is completely synthesized and secreted into the periplasm, where it is rapidly degraded. The complete synthesis of CutF and the dissociation of ribosomes prevent the unfolding of the downstream stem-loop, which shields the Shine-Dalgarno sequence (SD; red) of *cutO*. Consequently, the *cutFOG* mRNA is degraded. In the presence of Cu (lower panel), the nascent CutF protein binds Cu via its CxxxC motif, which transiently arrests the stalling sequence within the ribosomal tunnel. This provides a time window for the helicase activity of the ribosome to unfold the stem-loop and to initiate *cutO* translation via the now accessible SD. CutO is then produced and posttranslationally secreted via the Tat translocase into the periplasm, where it oxidizes Cu^1+^ to the less toxic Cu^2+^.

SecM is cotranslationally secreted by the SRP pathway and is rapidly degraded in the periplasm (45), which we also observe for CutF. In the absence of Cu, CutF is secreted into the periplasm and is degraded, as indicated by the observations that (i) CutF is only detectable in pulse-labeling experiments when the signal sequence is removed and (ii) the LepB-CutF fusion construct is proteolyzed in the absence of Cu. However, unlike SecM, the ribosome stalling-like sequence of CutF, *per se*, does not seem sufficient to achieve its translational stalling during export via the SecYEG translocon. This is seen with the LepB-CutF (SM10 or SM29) fusions, in which the SecM stalling sequence is substituted by the proline-rich (SM10 or SM29) sequences of CutF. This is also seen with the LepB-CutF fusion in the absence of Cu, despite the presence of the Cu-binding motif of CutF. However, in the presence of Cu, the translational stalling-like process that CutF undergoes is apparently prolonged, as is suggested by its decreased proteolysis due to the inaccessibility of its degradation sites caused by slowed translation. This occurs only when both the conserved Cu-binding and ribosome stalling-like motifs of CutF are present, as is seen with the LepB-CutF_C-A_ and LepB-CutF_ΔC-ter_ variants. The Cu-dependent, prolonged ribosomal stalling potentially unfolds the SL and renders the SD of *cutO* accessible for the initiation of translation (Fig. 8). Accordingly, Cu-dependent CutO production requires the presence of Cu, Cu-binding, and C-terminal proline-rich stalling-like motifs of CutF as well as its cotranslational export via a Sec signal sequence. As a corollary of this working model, in the absence of the SL, CutF and its characteristic features are dispensable, as is shown here.

Cu-induced ribosomal stalling also explains why most CutF-like proteins have a Sec signal sequence, as only a Sec signal sequence allows for the coupling of the CutF export to *cutFOG* translation. Further, the occurrence of a Sec signal sequence in CutF and Tat signal sequences in CutO and CutG is consistent with the observed physical contact between the SecYEG translocon and the Tat translocase (37, 46).

How Cu binding to CutF prolongs the translational stalling-like process seen in this work is currently unknown. A possibility could be the direct or indirect conformational changes of the nascent peptide, upon the binding of Cu, acting synergistically with the ribosomal stalling-like motif. Another possibility is that the folding force induced by Cu-binding to the CxxxC motif of CutF could be weaker than a disulfide-bridge formation and could thereby prolongate translational arrest (47). Alternatively, the short distance (45 amino acids) between the Cu-binding motif and the C-terminal motif may be critical. This positions the Cu-binding motif within the periplasmic vestibule of the SecY channel (31), assuming that approximately 30 amino acids are shielded within the ribosomal tunnel (48) and that approximately 17 amino acids are required to cross the bacterial membrane in an unbent conformation (49). The binding of Cu to the CxxxC motif of CutF, which is located in the confined space of the periplasmic vestibule of SecY, could restrict the entropic force acting on the translating polypeptide (50), thereby resulting in reduced translocation and prolonged translational arrest. Future structural studies depicting the exact conformation of CutF trapped in the ribosomal tunnel in the presence of Cu may further elucidate this process.

C-terminal stalling sequences that regulate the transcription of downstream genes have also been observed. One example is TnaC, which regulates tryptophan metabolism in E. coli. In the presence of tryptophan, the stalling sequence prevents the release of TnaC from the ribosome and inhibits rho-dependent transcription termination. Consequently, the transcription and the translation of the downstream-encoded TnaA and TnaB continue (51). Indeed, Cu-binding to the CutF-like protein B. pertussis CruR (SSN cluster 1) inhibits the production of downstream-encoded proteins via a ribosomal stalling-like process. Upon the binding of Cu, CruR triggers rho-dependent transcription termination, thereby preventing the production of the downstream-encoded TonB-dependent transporter (39). However, unlike R. capsulatus CutF (SSN cluster 15), CruR activity is Sec signal sequence-independent, its C-terminal, proline-rich, ribosomal stalling-like motif is slightly different, and the *cruR-bfgR* intergenic region is rather long (162 nucleotides) (39). Further, the occurrence of CutF-like proteins without the strict conservation of both the Sec signal peptide and the C-terminal, proline-rich, ribosomal stalling-like motifs are exemplified by the Cupriavidus metallidurans CzcI and CzcI2 lacking a C-terminal proline-motif (SSN cluster 20). CzcI is encoded upstream of the *czcCBA* RND efflux system and is suggested to act as a metal-sensing regulator (52). These observations illustrate that the specific modes of action of the different CutF-like proteins (SSN clusters 1 to 29) and the ensuing regulatory responses may be different and that their elucidation will require further studies.

In summary, in the presence of Cu, the ribosomal stalling-like motif, working together with the Cu-binding motif of CutF, can apparently delay its full-length synthesis long enough for ribosomes to unfold the downstream SL to allow for *cutO* translation. This enables cells to control CutO production in response to periplasmic Cu availability. The CutF-CutO pair serves as a fast-response by which to prevent Cu toxicity by allowing for the production of a periplasmic multicopper oxidase before the transcriptional regulation via the Cu-responsive cytoplasmic transcription factors, as with CueR or CopR (19, 20). The toxicity and abundance of Cu in natural environments (53, 54) likely justifies the small energetic investment of producing a low abundance and rapidly degraded CutF for boosting CutO production for palliating the toxicity of increasing Cu concentrations.

More broadly, this work shows that proteins containing C-terminal stalling sequences can sense intracellular metabolites, protein export defects as well as periplasmic and extracellular metabolite availability. Considering the abundance of CutF-like proteins and their close genetic associations with a myriad of proteins, this sensing mechanism is likely an important cornerstone for the adaptation of bacteria to changing environmental conditions.

## MATERIALS AND METHODS

### Bacterial strains, growth conditions, and plasmid construction.

The bacterial strains and plasmids used in this study are described in Table S1. R. capsulatus strains were grown under respiratory (Res) conditions on magnesium-calcium, peptone, yeast extract (MPYE) enriched medium (55) or on Sistrom’s minimal medium A (Med A) (56) supplemented with kanamycin, gentamicin, or tetracycline as appropriate (10, 1, or 2.5 μg per mL, respectively) at 35°C. For the arabinose-inducible genes in R. capsulatus, liquid media were supplemented with 0.5% l-arabinose (l-ara) at an OD_685_ of 0.5 to 0.6 and were further grown for 6 h. E. coli strains were grown on a lysogeny broth (LB) medium (57), containing ampicillin, kanamycin, or tetracycline (at 100, 50, or 12.5 μg per mL, respectively) as appropriate. The minimal medium M63 (18 amino acids), including all of the essential amino acids (with the exceptions of cysteine and methionine), was used for *in vivo* pulse-labeling experiments (37). Ampicillin at 50 μg per mL was used for the M63 medium.

10.1128/mbio.03040-22.8 TABLE S1 Strains and plasmids used in this work. Download Table S1, DOCX file, 0.03 MB. Copyright © 2023 Öztürk et al. 2023 Öztürk et al. https://creativecommons.org/licenses/by/4.0/ This content is distributed under the terms of the Creative Commons Attribution 4.0 International license.

### Cu sensitivity assays on plates.

The growth of the R. capsulatus strains in the absence or presence of 400 μM CuSO_4_ was monitored via streaking on plates or using spot assays (18). For the spot assays, strains were grown semi-aerobically overnight to an OD_685_ of approximately 0.9, and the cell counts were determined based on an OD_685_ of 1.0 = 7.5 × 10^8^ cells/mL. For each strain, 1 × 10^8^ cells were resuspended in 400 μL medium, and the cell suspension was subsequently serially diluted in a 96-well plate. Dilutions ranging from 10^0^ to 10^−7^ were spotted on MPYE plates by a 48-pin replica plater. The plates were incubated under the Res conditions for approximately 2 days before the data were scored.

### Construction of the p*cutF* and p*cutF*_Stp_*OG* plasmids.

The p*cutFOG* plasmid (15), carrying the tagged version of the *cutFOG* genes (*cutF*_N-Flag_*O*_C-Flag_*G*_C-MycHis_) and covering its 526 bp upstream (promoter) and 466 bp downstream (transcriptional terminator) parts, was used for genetic manipulations. The p*cutF* (*cutF*_N-Flag_ under the endogenous promoter) was constructed by removing the 280 bp *Srf*I fragment of p*cutFOG* that was covering the N-terminal 93 aa of the *cutO* gene which caused a frameshift mutation for the downstream parts of the operon. After the *Srf*I digestion and the gel purification of the large fragment of p*cutFOG* (Qiagen Gel Purification Kit; Qiagen, Hilden, Germany), T4 DNA ligase (NEB Lab, United States) was used for the self-ligation of the large fragment, following the manufacturer’s protocol. 5 μL of the ligation reaction was used for the transformation of the E. coli HB101 strain. The isolated plasmids from selected colonies were confirmed via sequencing. The p*cutF*_Stp_*OG* plasmid, carrying the start to stop codon substitution of *cutF* on p*cutFOG*, was constructed by using the cutF(EP)-F, cutFstop&noSS-R, cutFstop-F, and cutter-R primers (Table S2) to amplify the fragments covering the start to stop substitution mutation. After PCR amplification, the amplified products were treated with DpnI digestion to remove the template DNA and were purified using a Qiagen PCR Purification Kit (Qiagen, Hilden, Germany). Fragments containing the 20 bp overlapping sequences were assembled into the linearized (KpnI/XbaI digested) pRK415 plasmid via the NEBuilder^R^ HiFi assembly cloning method (NEB Lab, United States), following the manufacturer’s protocol. The total amount of DNA fragments used was approximately 0.4 to 0.5 pmol, and the vector to insert ratio was approximately 1:2. The samples were incubated in a thermocycler at 50°C for 60 min, and 4 μL of the assembly reaction was transformed to a chemically competent E. coli HB101 strain. The pPara-*cut*O plasmid was constructed by using the 1F-92NOQ/Pbad-R primer pair to amplify the *araC*-Promoter_Ara_ fragment from the pBAD plasmid and the cutO-F/cutO-R primer pair to amplify the *cutO*_C-Flag_ from the genomic DNA of R. capsulatus (15).

10.1128/mbio.03040-22.9 TABLE S2 Primers used in this work (5′ to 3′ direction). Download Table S2, DOCX file, 0.01 MB. Copyright © 2023 Öztürk et al. 2023 Öztürk et al. https://creativecommons.org/licenses/by/4.0/ This content is distributed under the terms of the Creative Commons Attribution 4.0 International license.

### Construction of the SL mutated derivatives of the *cut* operon without *cutF*.

The constructions of p*cut(+SL)OG*, p*cut(−SL)OG*, and p*cut(SLm)OG* (Fig. S2A) were performed by using the cutO(+SL)G-F/cutTer-R, cutO(−SL)G-F/cutTer-R, and cutO(SLm)G-F/cutTer-R primer pairs, respectively (Table S2). The promoter region was amplified by using the cutF(EP)-F/cutF(EP)-R primer pairs (Table S2). The amplified fragments were subjected to DpnI digestion to remove the template DNA (p*cutFOG*) and were purified using a Qiagen PCR Purification Kit (Qiagen, Hilden, Germany). Fragments were integrated into the linearized (KpnI/XbaI digested) pRK415 plasmid via the NEBuilder^R^ HiFi assembly cloning method (NEB Lab, United States), as described above.

### Construction of the signal peptide and stem-loop manipulated CutF derivatives.

The signal peptide manipulated CutF derivatives p*cutF_Δ_*_SP_*OG* and p*cutF*_Tat-NosZ_*OG* (Table S1) were constructed by using the primer pairs cutF(EP)-F/cutFstop&noSS-R and cutFnoSS-F/cutFter-R for p*cutF_Δ_*_SP_*OG* as well as cutF(EP)-F/cutF-Tat-R and cutF-Tat-F/cutFter-R for p*cutF*_Tat-NosZ_*OG* (Table S2). Amplified fragments carrying the desired modifications were cloned to a linearized pRK415 plasmid, as described above. The stem-loop mutation (SLm; CTTC to AAAA mutation of a-SD in the stem-loop) was introduced to p*cutFOG*, p*cutF*_C-A_*OG*, p*cutF_Δ_*_C-Ter_*OG*, and p*cutF_Δ_*_SP_*OG* by using the cutO-SL-F/cutO-SL-R primer pair (Table S2).

The correct constructs on plasmid pRK415 were conjugated into the corresponding R. capsulatus strains (*ΔcutF* for p*cutF* and *ΔcutFOG* for the other constructed plasmids) via triparental conjugation (58) (Table S1).

### Cloning of the CutF and LepB-CutF variants to the *T7*-based expression plasmid pRS1 for the *in vivo* pulse-labeling, *in vitro* expression, and cross-linking experiments.

The plasmid pRS1-CutF that was used for the *in vivo* and *in vitro* experiments was described previously (15). For the construction of the signal peptide and the C-terminal deletion versions of CutF in pRS1 (pRS-CutF_ΔSP_ and pRS-CutF_ΔC-ter_, respectively), a Q5 Mutagenesis Kit (NEB Lab, MA) was used with the mutagenic primer pairs pRScutF(noSS)-F/pRS1-R for pRS-CutF_ΔSP_ and pRS1-F/pRScutF(delC-ter)-R for pRS-CutF_ΔC-ter_ (Table S2). The pRSLepB–CutF(SM10) and pRSLepB–CutF(SM28) were constructed using a Q5 Mutagenesis Kit (NEB Lab, MA) with the cutF(AP 10&28)-F/cutF(AP-10aa)-R and cutF(AP 10&28)-F/cutF(AP-28aa)-R mutagenic primer pairs carrying the 10 and 28 aa C-terminal, proline-rich segment of CutF, respectively (Fig. 4B; Table S2). 10 ng of pRS1-LepB–SecM(Ms) (37) was used as a template, and the manufacturer’s protocol was followed. 5 μL of the KLD reaction mixture was transformed to a chemically competent E. coli NEB 5-alpha strain that was selected for the Amp^R^ colonies. The resulting plasmids carrying new constructs were confirmed via DNA sequencing and were transformed to MC4100 via the TSB transformation procedure (59). For the LepB-CutF fusion construct, pRSLepB–CutF(SM10) was linearized by using the cutF(AP10&28)-F/LepB-cutF-V-R primer pairs via inverse PCR to keep the first and second TM helix and the last 23 aa of lepB, excluding the rest of the sequence. The *cutF*, excluding the signal peptide and including a 20 bp overlapping sequence of the *lepB* second TM helix at the 5′ end and the last 23 aa seq at the 3′ end, was amplified from p*cutFOG* by using the primers cutF(LepB)-F and cutF(LepB)-R (Table S2). The PCR products were digested with DpnI to remove the template DNA used for the PCR and were then purified using a Qiagen PCR Purification Kit (Qiagen, Hilden, Germany). The NEBuilder^R^ HiFi assembly cloning method (NEB Lab, United States) and transformation procedures similar to those described above were used for this construction. The resulting plasmid pRSLepB-CutF(fusion) was confirmed via DNA sequencing and was transformed to MC4100 via the TSB transformation procedure (60). The LepB-CutF_C-A_ and LepB-CutF_ΔC-ter_ versions were constructed using a Q5 Mutagenesis Kit (NEB Lab, MA) with the cutF(CtoA)-F/cutF(CtoA)-R and lepB-cutF(dC-ter)-F/lepB-cutF(dC-ter)-R mutagenic primer pairs carrying the CxxxC to AxxxA and Δ10 amino acids from the C-terminal, proline-rich segment, respectively (Table S2). 10 ng of pRS-LepB–CutF was used as a template, and the manufacturer’s protocol was followed. 5 μL of the KLD reaction mixture was transformed to a chemically competent E. coli NEB 5-alpha strain that was selected for the Amp^R^ colonies. The resulting plasmids were confirmed via DNA sequencing and were transformed to MC4100 via the TSB transformation procedure (59).

### Preparation of the periplasmic fraction for multicopper oxidase activity assays and SDS-PAGE.

The periplasmic fraction from R. capsulatus cells was isolated from 50 mL overnight (approximately 20 h) cultures and grown on MPYE medium under the Res conditions at 35°C with 110 rpm shaking in the absence or presence of 10 μM CuSO_4_ (60, 61). Cells were harvested and washed at 4°C with 12 mL of 50 mM Tris-HCl pH 8.0. The pellet was resuspended to a concentration of 10 mL/g of wet weight in SET buffer (0.5 M sucrose, 1.3 mM EDTA, 50 mM Tris-HCl pH 8.0) and was incubated with 600 μg lysozyme/mL at 30°C for 60 min. The formation of spheroplasts was monitored via microscopy. Spheroplasts were collected via centrifugation (13,000 rpm) at 4°C for 30 min. The supernatant (periplasmic fraction) was either directly used for multicopper oxidase activity assays and SDS-PAGE or stored at −80°C.

The oxidation of 2,6-dimethoxyphenol (2,6-DMP) by the periplasmic fraction (50 μg total protein) was monitored as an endpoint assay at 468 nm. The molar concentration of oxidized 2,6-DMP was calculated using ɛ = 14,800 M^−1 ^cm^−1^ (62).

### Immune detection.

Following the SDS-PAGE, proteins were electroblotted onto nitrocellulose (GE Healthcare, Germany) or PVDF Immobilon-P (GE Healthcare, Germany) membranes, and antibodies against the Flag-tags were purchased from either Sigma or Millipore (Temecula, USA).

### *In vitro* synthesis and cross-linking of CutF.

*In vitro* protein synthesis was performed in an E. coli *in vitro* transcription/translation system, as described before (32). Samples were incubated with gentle shaking at 37°C for 30 min. The reaction was stopped with 5% trichloroacetic acid (TCA) for 30 min on ice. Precipitated proteins were pelleted via centrifugation (13,000 rpm) and were resuspended in 30 μL TCA loading dye (63). Samples were separated on a 5 to 15% SDS-PAGE and were visualized via phosphorimaging.

For the cross-linking of the *in vitro* synthesized CutF, the triethanolamine acetate (pH 7.5) was replaced with HEPES/NaOH (pH 7.5) buffer in the *in vitro* reaction. 36 ng/μL of purified SRP or SecA, both in 50 mM HEPES/NaOH (pH 7.5), 100 mM K acetate, 10 mM Mg acetate, and 1 mM DTT, were added to the *in vitro* reaction and incubated with gentle shaking at 37°C for 30 min. The SRP was reconstituted from purified Ffh and 4.5S RNA (63), and the SecA was purified as described (64). Subsequently, 7.5 μL 25 mM DSS (Thermo Fisher, Germany) dissolved in dimethyl sulfoxide was added to each reaction. The samples were incubated at 25°C for 30 min and were then quenched with 50 mM Tris/HCl (pH 7.5). Subsequently, proteins were TCA precipitated and visualized, as described above.

### *In vivo* pulse-labeling.

The E. coli MC4100 strain carrying the appropriate plasmids was grown at 37°C overnight in 3 mL LB medium containing 100 μg/mL ampicillin. Cultures (2 mL) were harvested and resuspended after two washing steps in 200 μL of M63 medium (18 aa). 150 μL of the cell suspensions were used for the inoculation of 10 mL fresh M63 medium supplemented with 25 μg/mL ampicillin. The cultures were grown at 37°C with 180 rpm shaking until an OD_600_ value of 0.5 to 0.8 was reached. Subsequently, 2 × 10^8^ cells were collected and transferred to 2-mL Eppendorf tubes, and the volume was adjusted to 2 mL with fresh M63 medium. 50 μg/mg rifampicin was added, and this was followed by incubation for 15 min at 37°C. The production of the LepB–SecM and LepB–CutF variants was induced by the simultaneous addition of 0.1 M IPTG and 2 μL of l-[^35^S] methionine-cysteine (7 mCi/mL, PerkinElmer Life Sciences) (37). CCCP (0.1 mM) was added when indicated, and the cultures were preincubated for 10 min at 37°C prior to the addition of IPTG and l-[^35^S] methionine-cysteine. CuSO_4_ was added together with IPTG and l-[^35^S] methionine-cysteine. Subsequently, 100 μL of each sample were collected after different time points and directly precipitated via the addition of 10% TCA and incubation on ice for 30 min. The precipitated proteins were pelleted by 15 min of centrifugation at 13,500 rpm at 4°C. The pellets were denatured in 25 μL SDS-loading dye at 56°C for 15 min with continuous shaking at 1,400 rpm. The samples were separated by SDS-PAGE (15%) and were analyzed via phosphorimaging (37).

### Bioinformatic analyses.

To generate a list of proteins that share characteristics with CutF, we used a previously generated list of CutF-like proteins that relied on a set of rules (i.e., searched for small ORFs within a 10 gene window of the gene encoding a CutO-like protein [defined as containing PF00394, PF07731, or both], encoded protein is smaller than 170 aa, does not match to an annotated Pfam domain, contains a signal peptide, contains CxxxC, contains a PP motif, limited to Proteobacteria) (15). A multiple sequence alignment of the proteins from this list was generated using MUSCLE (65), and it was used to search against the UniProt database (Reference Proteomes) with jackhammer (using 6 iterations) (66). This list, named list B in Fig. 6, was filtered based on length (proteins longer than 150 amino acids were removed), the presence of a PP motif within 15 amino acids of the C terminus, the presence of a signal peptidase I-cleaved Sec signal peptide (as predicted by SignalP) (67), whether the neighboring downstream gene was on the same strand, and whether the intergenic region with that neighboring gene consisted of fewer than 1,000 nucleotides. A sequence similarity network was built with these sequences, using an E value threshold of 1E–5 (i.e., an alignment score of 5) and the EFI-EST webtool (68). The network was visualized with Cytoscape v3.5.0 (69) and the yFile Organic layout. The clusters containing more than 10 nodes were then used to start separate jackhammer searches (i.e., the sequences in each cluster were aligned and used as a query for a jackhammer search against UniProt [Reference Proteomes] with 5 iterations). The proteins resulting from each jackhammer search were combined (resulting in list C) and were filtered using the same filters as were used with list B (except that putative lipoprotein signal peptides transported by the Sec translocon and cleaved by Signal Peptidase II, which were detected for CruR, were included in addition to the “standard” secretory signal peptides), thereby creating list D. Genomic context information was collected using the EFI-GNT webtool (70).
