# Supplementary figures and images for: Metabolic Sensing of Extracytoplasmic Copper Availability via Translational Control by a Nascent Exported Protein
Source: mBio. 2023 Jan 4;14(1):e03040-22. doi: 10.1128/mbio.03040-22 (PMC9973294; doi:10.1128/mbio.03040-22)

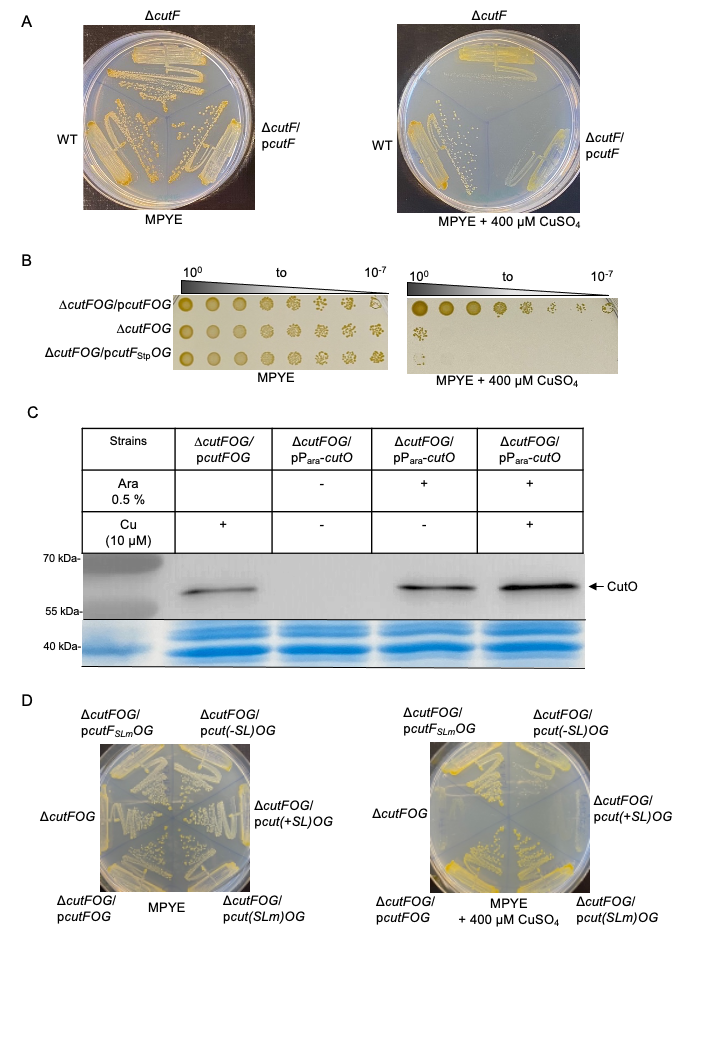

Supplement: FIG S1 [file mbio.03040-22-s0001.tif]

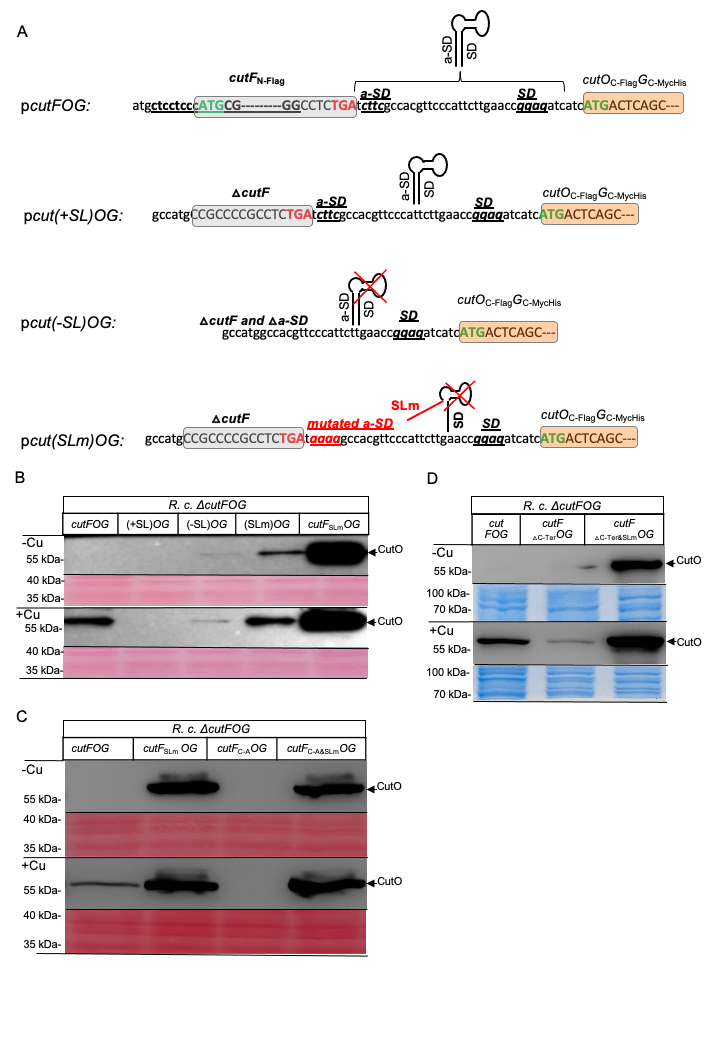

Supplement: FIG S2 [file mbio.03040-22-s0002.tif]

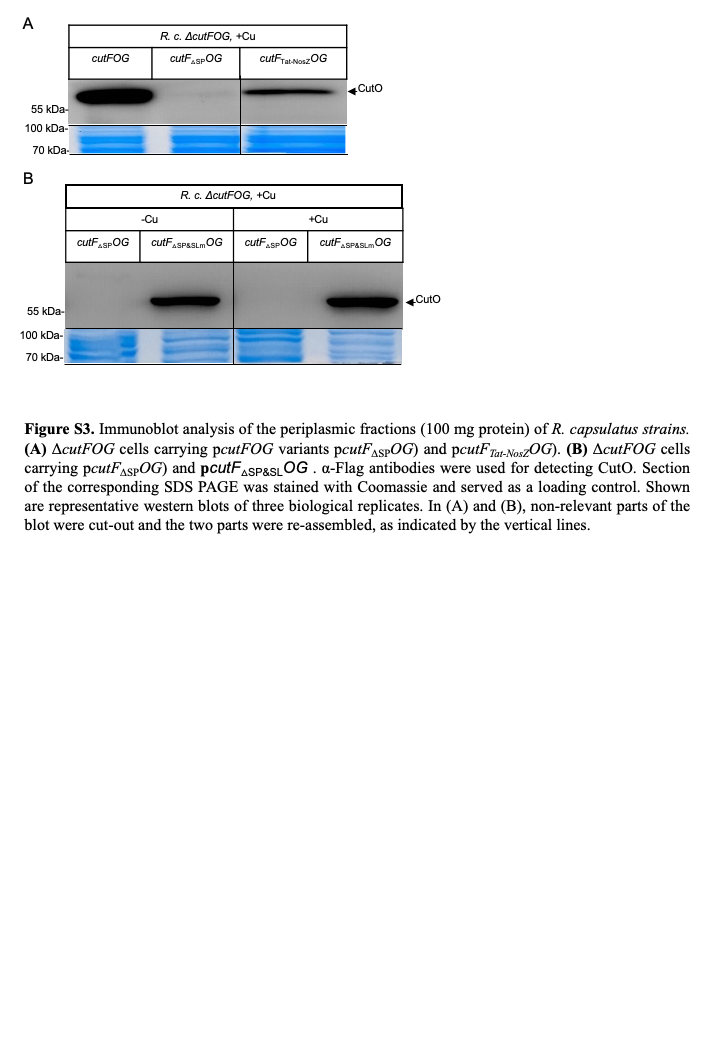

Supplement: FIG S3 [file mbio.03040-22-s0003.tif]

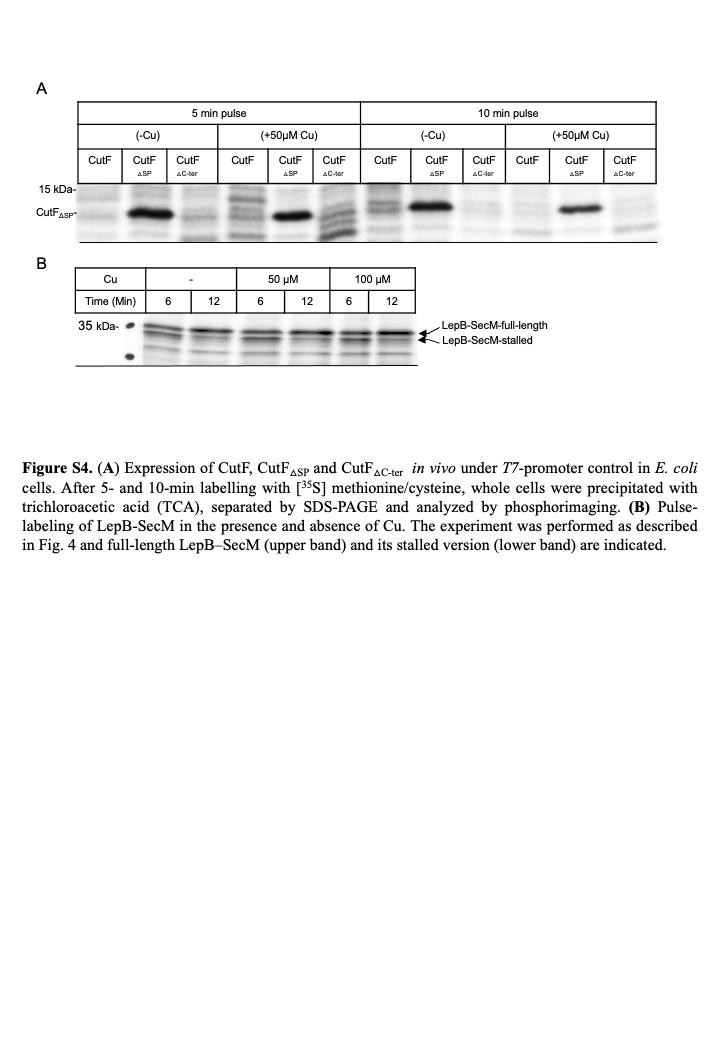

Supplement: FIG S4 [file mbio.03040-22-s0004.tif]

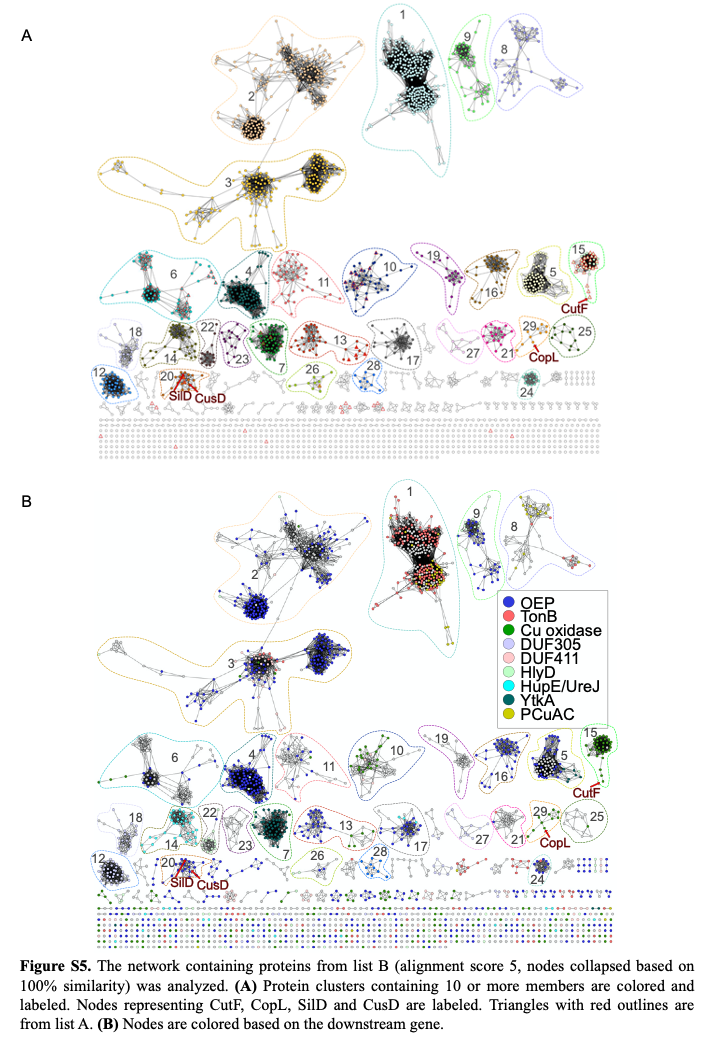

Supplement: FIG S5 [file mbio.03040-22-s0005.tif]

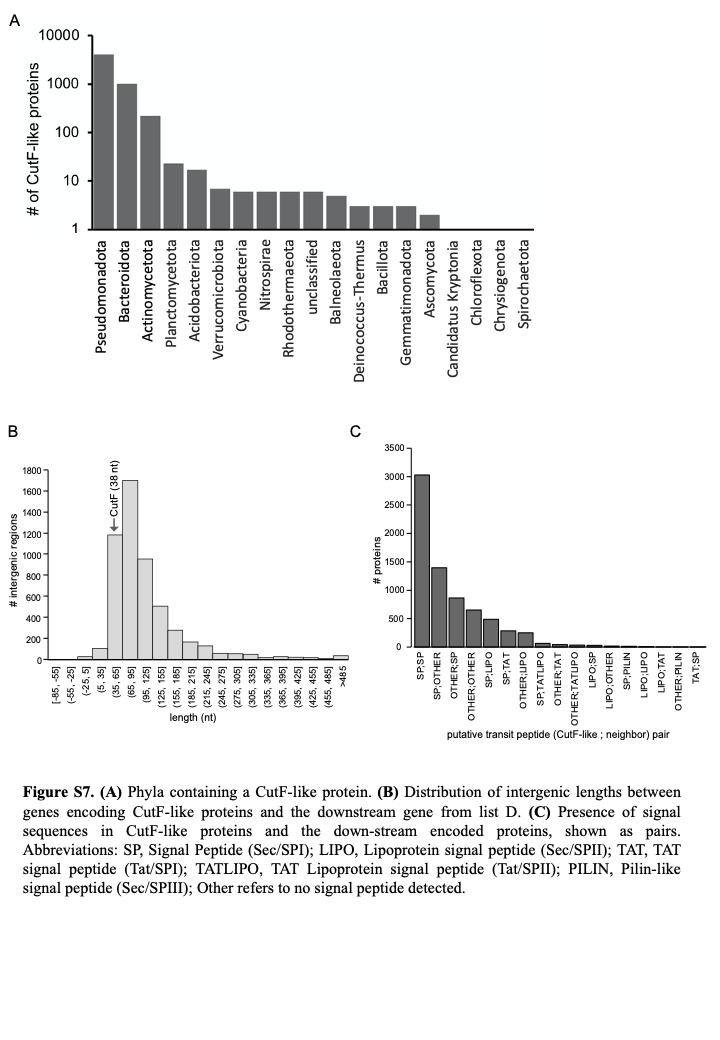

Supplement: FIG S7 [file mbio.03040-22-s0007.tif]
