# Supplementary material for: Metabolic Sensing of Extracytoplasmic Copper Availability via Translational Control by a Nascent Exported Protein
Source: mBio. 2023 Jan 4;14(1):e03040-22. doi: 10.1128/mbio.03040-22 (PMC9973294; doi:10.1128/mbio.03040-22)
Supplement: FIG S6 [file mbio.03040-22-s0006.pdf]

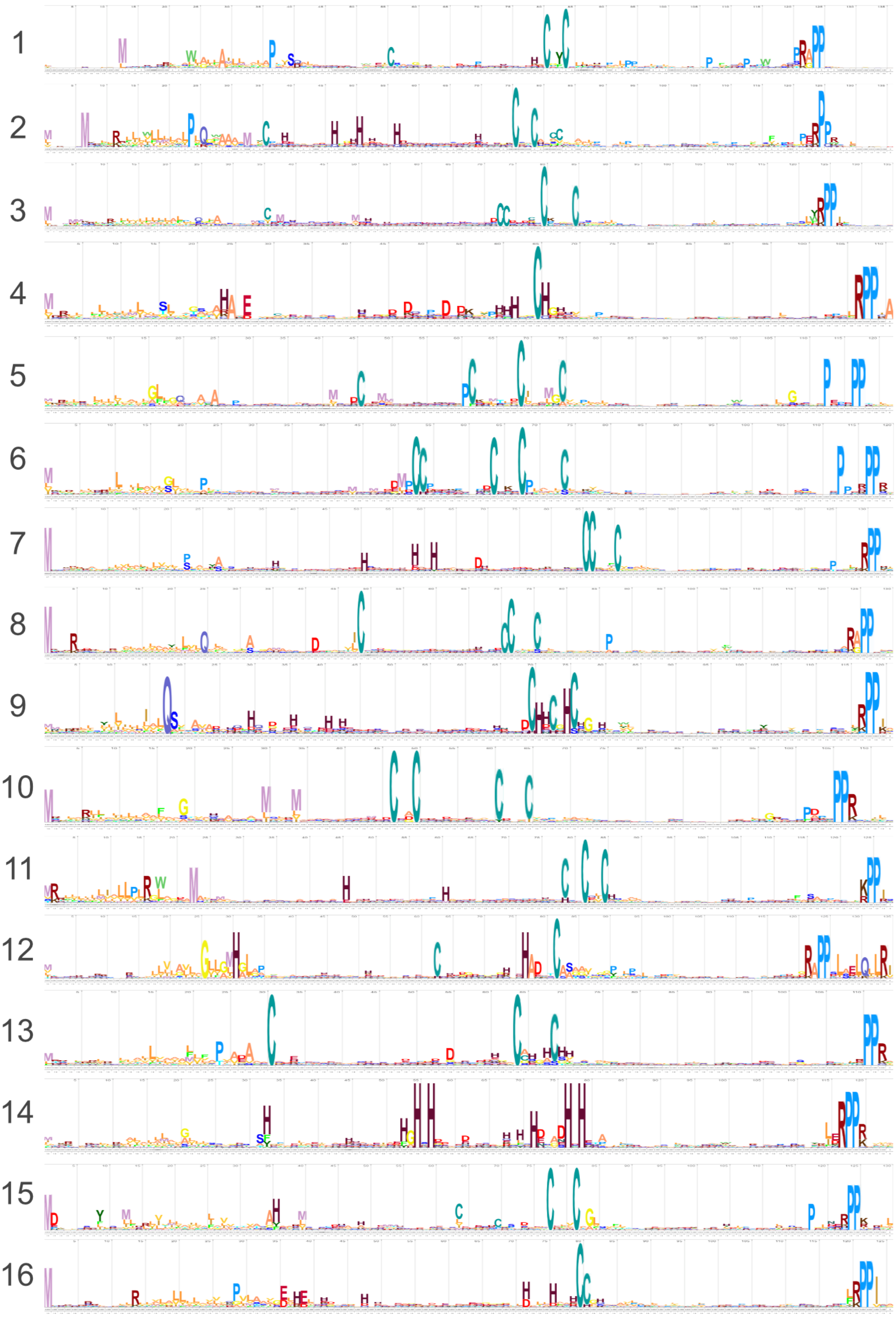

Figure S6. Sequence logos representing profile-HMM generated with list B clusters. The number to the right refers to the cluster number in Figure 9. The "CutF" logo represents the profile-HMM from a search using CutF against UniProt using phmmer followed by jackhammer for 5 iterations.

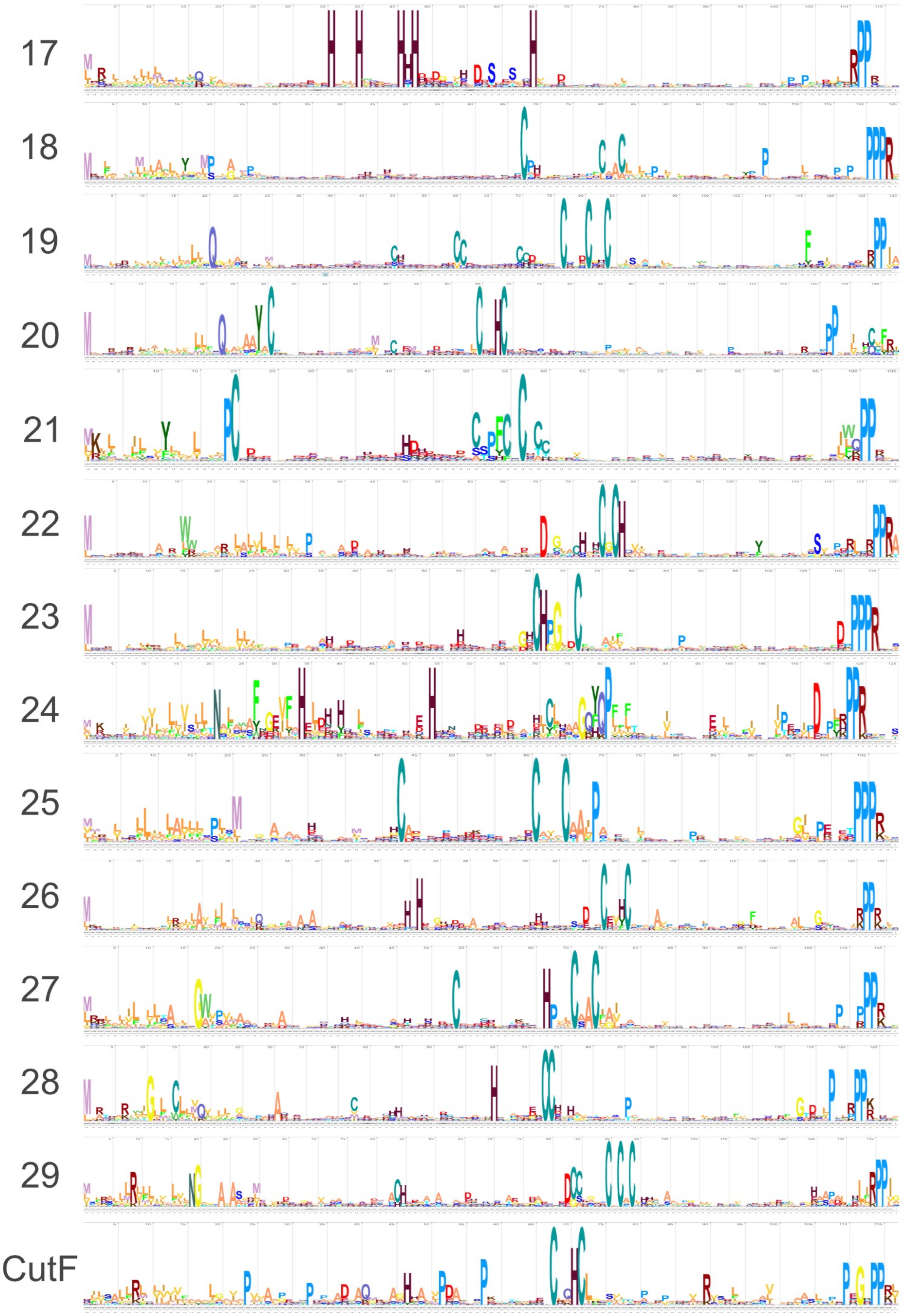

Figure S6. continued
