# Supplementary material for: Metabolic Sensing of Extracytoplasmic Copper Availability via Translational Control by a Nascent Exported Protein
Source: mBio. 2023 Jan 4;14(1):e03040-22. doi: 10.1128/mbio.03040-22 (PMC9973294; doi:10.1128/mbio.03040-22)
Supplement: TABLE S1 [file mbio.03040-22-s0008.docx]

**Table S1.** Strains and plasmids used in this work.

| **Strain or plasmid** | **Description** | **Phenotype** | **Reference** |
| --- | --- | --- | --- |
| ***Strains***  ***E. coli*** |  |  |  |
| HB101 | F^-^ Δ(*gpt-proA*)62 *leuB6 supE44 ara*-14 *galK2 lacY*1  Δ(*mcrC-mrr*) *rpsL20* (Str^R^) *xyl-5 mtl-1recA*13 | Str^r^ | (1) |
| NEB 5-α | ﻿fhuA2 D(argF-lacZ)U169 phoA glnV44 f80D(lacZ)M15 gyrA96 recA1  relA1 endA1 thi-1 hsdR17 |  | ﻿NEB #C2987 |
| MC4100 | Wild type strain |  | (2) |
| ***R. capsulatus*** |  |  |  |
| ^a^MT1131 | *crtD121* | wild type (Rif^r^) | (3) |
| Δ*cutF*  (YO-ΔcutF) | Δ( *cutF*(*rcc02111*)) seamless in frame deletion | In-frame, markerless | (4) |
| Δ*cutFOG*  (YO-ΔcutFOG) | Δ ( *rcc02111*)*,* Δ(*rcc03065-rcc03067::Gm*), Δ(*cutO::Kan*) | Gm^r^ | (5) |
| ***Plasmids*** |  |  |  |
| pRK2013 | Conjugation helper | Kan^r^ | (6) |
| pRK415 | Broad host-range vector | Tet^r^ | (6) |
| pRS1 | pPET19 based containing pBR322 ori, Rop, T7RNAP under EM7 promoter, LacI | Amp^r^ | (7) |
| pP_ara_-*cutO*  (pYO-cutO_Flag_) | *cutO*_Flag_ under the arabinose promoter (P_ara_) in pRK415 | Tet^r^ | (5) |
| p*cutFOG*  (pRK-cutFOG3) | *cutF*_N-ter_ _Flag_, *cutO*_Flag_ and  *cutG*_MycHis_ with promoter and terminator regions of *cutFOG* operon on pRK415 | Tet^r^ | (5) |
| p*cutF*_C-A_*OG*  (pRK-cutF_C-A_OG3) | substitution of conserved Cys to Ala of CutF (*C_69_XXXC_73_* to *A_69_XXXA_73_* ) on p*cutFOG* | Tet^r^ | (5) |
| p*cutF*_∆C-ter_*OG*  (pRK-cutF_∆C-ter_OG3) | truncation of C-terminus Pro-rich region of CutF (∆P_109_EPEGPPPRL_118_: 10 aa) on p*cutFOG* | Tet^r^ | (5) |
| p*cutF* | *cutF*_N-Flag_ with promoter and terminator regions of  *cutFOG* operon on pRK415. 280 bp SrfI fragment of p*cutFOG* covering the N-terminus 93 aa of the *cutO* gene was removed. | Tet^r^ | This work |
| p*cutF*_Stp_*OG* | Conversion of *cutF* start codon ATG to stop codon TAG on p*cutFOG*. | Tet^r^ | This work |
| p*cutF*_SLm_*OG* | CTTC to AAAA mutation on anti-SD of Steem-loop (SLm) between the *cutF-CutO* intergenic region on p*cutFOG*. | Tet^r^ | This work |
| p*cutF*_C-A&SLm_*OG* | combination of  *C_69_XXXC_73_* to *A_69_XXXA_73_* substitution of CutF and Steem-loop mutation (CTTC to AAAA substitution) between the *cutF-CutO* intergenic region on p*cutFOG*. | Tet^r^ | This work |
| p*cutF*_∆C-ter&SLm_*OG* | combination of the CutF truncation at C-terminus (∆P_109_EPEGPPPRL_118_: 10 aa) and Steem-loop mutation (CTTC to AAAA substitution) between the *cutF-CutO* intergenic region on p*cutFOG*. | Tet^r^ | This work |
| p*cut(+SL)OG* | erasing *cutF* from *cutFOG* operon on p*cutFOG*. | Tet^r^ | This work |
| p*cut(-SL)OG* | erasing *cutF* and anti-SD of Steem-loop between the *cutF-CutO* intergenic region on p*cutFOG*. | Tet^r^ | This work |
| p*cut(SLm)OG* | erasing *cutF* and CTTC to AAAA mutation on anti-SD of Steem-loop between the *cutF-CutO* intergenic region on p*cutFOG*. | Tet^r^ | This work |
| p*cutF*_∆SP_ *OG* | truncation of Sec signal peptide of CutF on p*cutFOG*. | Tet^r^ | This work |
| p*cutF*_Tat-NosZ_*OG* | Replacing SP of CutF with Tat SP of NosZ  (MTEETRSGISRRMLLGATAGGAALAGGLAGRLALGTGIGAATLATAAQ) | Tet^r^ | This work |
| p*cutF*_∆SP&SLm_ *OG* | combination of the SP truncated version of CutF and Steem-loop mutation (CTTC to AAAA substitution) between the *cutF-CutO* intergenic region on p*cutFOG*. | Tet^r^ | This work |
| pRS-CutF | *cutF*_N-Flag_ ORF cloned to pRS1 for in vitro expression | Amp^r^ | (5) |
| pRS-CutF_∆SP_ | truncation of Sec signal peptide of CutF_N-Flag_ on pRS1 | Amp^r^ | This work |
| pRS-CutF_∆C-ter_ | truncation of C-terminus Pro-rich region of CutF_N-Flag_ (∆P_109_EPEGPPPRL_118_: 10 aa) on pRS1 | Amp^r^ | This work |
| pRS1-LepB-SecM(Ms) | arrest peptide of SecM ( HAPIRGSP) from ﻿*M. succiniciproducens* was inserted at the C-terminus of LepB from *E. coli* | Amp^r^ | (7) |
| pRSLebB-CutF(SM:10aa) | SecM arrest peptide(8aa) was replised by 10 aa of C-ter of CutF (P_109_EPEGPPPRL_118_) on pRSLeb-secM(Ms)-L=63 | Amp^r^ | This work |
| pRSLebB-CutF(SM:28aa) | SecM arrest peptide(8aa) was replised by 10 aa of C-ter of CutF  ( R_91_ISRLVPPAVPLRLALLSPEPEGPPPRL_118_) on pRSLeb-secM(Ms)-L=63, | Amp^r^ | This work |
| pRSLebB-CutF | ORF of *cutF* (excluding SP) fused to LepB after 2^nd^ transmembrane helix | Amp^r^ | This work |
| pRSLebB-CutF_C-A_ | substitution of conserved Cys to Ala of CutF (*C_69_XXXC_73_* to *A_69_XXXA_73_* ) on pRSLebB-CutF | Amp^r^ | This work |
| pRSLebB-CutF_∆C-ter_ | truncation of C-terminus Pro-rich region of CutF_N-Flag_ (∆P_109_EPEGPPPRL_118_: 10 aa) on pRSLebB-CutF | Amp^r^ | This work |

^a^*R.capsulatus* strain MT1131 is derived from SB1003 in multiple steps, as described in (3): first a Ps-deficient mutant (TL1) was obtained using tetracycline suicide, then its *crtD* derivative was constructed by GTA cross to yield MT113, and then its Ps-proficient derivative was obtained via a second GTA cross.

**SI References**

1. Sambrook J, Russell DW. 2001. Molecular Cloning: a laboratory manual, 3rd ed. ed. Cold Spring harbor Laboratory Press, Cold Spring harbor.

2. Götzke H, Palombo I, Muheim C, Perrody E, Genevaux P, Kudva R, Müller M, Daley DO. 2014. YfgM is an ancillary subunit of the SecYEG translocon in *Escherichia coli*. Journal of Biological Chemistry 289:19089-19097.

3. Zannoni D, Prince RC, Dutton PL, Marrs BL. 1980. Isolation and Characterization of a Cytochrome *c*_2_-Deficient Mutant of *Rhodopseudomonas-capsulata*. Febs Letters 113:289-293.

4. Selamoglu N, Önder Ö, Öztürk Y, Khalfaoui-Hassani B, Blaby-Haas CE, Garcia BA, Koch H-G, Daldal F. 2020. Comparative differential cuproproteomes of *Rhodobacter capsulatus* reveal novel copper homeostasis related proteins. Metallomics 12:572-591.

5. Öztürk Y, Blaby-Haas CE, Daum N, Andrei A, Rauch J, Daldal F, Koch HG. 2021. Maturation of *Rhodobacter capsulatus* Multicopper Oxidase CutO Depends on the CopA Copper Efflux Pathway and Requires the cutF Product. Front Microbiol 12:720644.

6. Ditta G, Schmidhauser T, Yakobson E, Lu P, Liang XW, Finlay DR, Guiney D, Helinski DR. 1985. Plasmids related to the broad host range vector, pRK290, useful for gene cloning and for monitoring gene expression. Plasmid 13:149-53.

7. Jauss B, Petriman NA, Drepper F, Franz L, Sachelaru I, Welte T, Steinberg R, Warscheid B, Koch HG. 2019. Noncompetitive binding of PpiD and YidC to the SecYEG translocon expands the global view on the SecYEG interactome in *Escherichia coli*. Journal of Biological Chemistry 294:19167-19183.
