# Supplementary material for: Metabolic Sensing of Extracytoplasmic Copper Availability via Translational Control by a Nascent Exported Protein
Source: mBio. 2023 Jan 4;14(1):e03040-22. doi: 10.1128/mbio.03040-22 (PMC9973294; doi:10.1128/mbio.03040-22)
Supplement: TABLE S2 [file mbio.03040-22-s0009.docx]

##### Table S2. Primers used in this work (5’ to 3’ direction).

| Pbad-R | 5-catggttaattcctcctgttagcc-3 |
| --- | --- |
| cutF(EP)-F | 5­agtgaattcgagctcggtacatcctgcgcctgaaaggccag-3 |
| cutTer-R | 5-tgcatgcctgcaggtcgactaatagtctctttggcctgctgccg-3 |
| cutFstop-F | 5-ccccgtgccatgctcctccctagcggattctgtgcgccctgcttc-3 |
| cutFstop&noSS-R | 5-gggaggagcatggcacggggcgggg -3 |
| cutFnoSS-F | 5-tgccatgctcctcccatggcggactacaaggacgacgatgacaagggcggc-3 |
| cutO-SL-F | 5-gccgccgccccgcctctgataaaagccacgttcccattcttgaacc-3 |
| cutO-SL-R | 5-atcagaggcggggcggcggcccctcc-3 |
| cutF-Tat-F | 5-tggcgggcgggcttgccggccgtctggcgcttggcaccgggatcggcgcggcgacgctggc caccgcggcgcaggccgcggactacaaggacgacgatgacaagggcggctc-3 |
| cutF-Tat-R | 5-gccggcaagcccgcccgccagcgccgcgccgcccgcggtggcgcccagaagcatcctgcgg ctgatgccggatctggtttcctcggtcatgggaggagcatggcacggggc-3 |
| cut(-SL)OG-F2 | 5-ccccgtgccatggccacgttcccattcttgaaccggagatcatc-3 |
| cutF(EP)-R | 5-catggcacggggcggggccgttg-3 |
| cutO(+SL)G-F | 5­ccccgtgccatgccgccccgcctctgatcttcgccacgttcccattc-3 |
| cutO(SL Mut)G-F | 5-ccccgtgccatgccgccccgcctctgataaaagccacgttcccattcttgaaccggag-3 |
| pRS1-F | 5-gcggccgcgagctcgtcgacctcgagtagc-3 |
| pRS1-R | 5-catggggtatatctccttcttaaagttaaacaaaattatttc-3 |
| pRS1cutF-R | 5-gtcgacgagctcgcggccgctcagaggcggggcggcggcccctcc-3 |
| pRScutF(noSS)-F | 5-gcggactacaaggacgacgatgacaagg-3 |
| pRScutF(delC-ter)-R | 5­tcaggaaagcagcgccagccgcaacg-3 |
| cutF(AP 10&28)-F | 5-ccggaaccggaggggccgccgccgcgcctcgggagctccgataagcaagaaggc-3 |
| cutF(AP-10aa)-R | 5-ggagctcatccagatagccgttgc-3 |
| cutF(AP-28aa)-R | 5-ggaaagcagcgccagccgcaacggaaccgccggcggaacaaggcgcgagatgcgggag ctcatccagatagccgttgc-3 |
| cutF(lepB)-F | 5-tgattgtgcgttcgtttattgcgccggaggcctgtccgcatccc-3 |
| LepB-cutF-V-R | 5-aataaacgaacgcacaatcaatacgatagccagtaccg-3 |
| cutF(lepB)-R | 5-ggcggcccctccggttccggggaaagc-3 |
| cutF(CtoA)-F | 5-gcgctgcagcatgcgctgggcgcctcgctgacgcccg-3 |
| cutF(CtoA)-R | 5-gccttccggcgcgaaggtggtggtcttgg-3 |
| lepB-cutF(dC-ter)-F | 5-gggagctccgataagcaagaaggcgaatg-3 |
| lepB-cutF(dC-ter)-R | 5-ggaaagcagcgccagccgcaacg-3 |
